# *IKZF1* and *UBR4* gene variants drive autoimmunity and Th2 polarization in IgG4-related disease

**DOI:** 10.1172/JCI178692

**Published:** 2024-06-13

**Authors:** Qingxiang Liu, Yanyan Zheng, Ines Sturmlechner, Abhinav Jain, Maryam Own, Qiankun Yang, Huimin Zhang, Filippo Pinto e Vairo, Karen Cerosaletti, Jane H. Buckner, Kenneth J. Warrington, Matthew J. Koster, Cornelia M. Weyand, Jörg J. Goronzy

**Affiliations:** 1 Department of Immunology,; 2 Department of Medicine,; 3 Center for Individualized Medicine and Department of Clinical Genomics, Mayo Clinic College of Medicine and Science, Rochester, Minnesota, USA.; 4 Center for Translational Immunology, Benaroya Research Institute at Virginia Mason, Seattle, Washington, USA.

**Keywords:** Autoimmunity, T cell receptor, Th2 response, Tolerance

## Abstract

IgG4-related disease (IgG4-RD) is a systemic immune-mediated fibroinflammatory disease whose pathomechanisms remain poorly understood. Here, we identified gene variants in familial IgG4-RD and determined their functional consequences. All 3 affected members of the family shared variants of the transcription factor IKAROS, encoded by *IKZF1*, and the E3 ubiquitin ligase UBR4. The IKAROS variant increased binding to the *FYN* promoter, resulting in higher transcription of *FYN* in T cells. The *UBR4* variant prevented the lysosomal degradation of the phosphatase CD45. In the presence of elevated FYN, CD45 functioned as a positive regulatory loop, lowering the threshold for T cell activation. Consequently, T cells from the affected family members were hyperresponsive to stimulation. When transduced with a low-avidity, autoreactive T cell receptor, their T cells responded to the autoantigenic peptide. In parallel, high expression of FYN in T cells biased their differentiation toward Th2 polarization by stabilizing the transcription factor JunB. This bias was consistent with the frequent atopic manifestations in patients with IgG4-RD, including the affected family members in the present study. Building on the functional consequences of these 2 variants, we propose a disease model that is not only instructive for IgG4-RD but also for atopic diseases and autoimmune diseases associated with an *IKZF1* risk haplotype.

## Abstract 2

In familial IgG4-related disease, *IKZF1* and *UBR4* gene variants break T cell tolerance and concomitantly induce a Th2 response.

## Graphical abstract

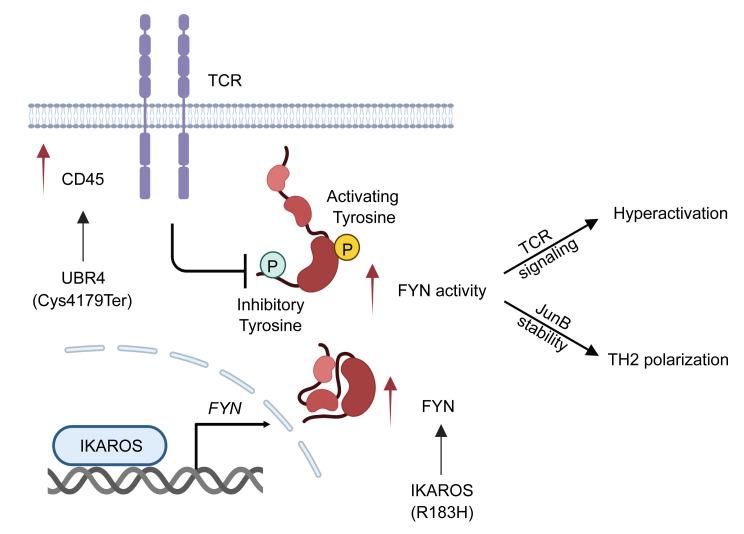

## Introduction

Most autoimmune diseases have a genetic component, with only a few demonstrating monogenic inheritance. Variants in more than 100 genes have been reported to contribute to genetic predisposition to autoimmune diseases (1). Drawing conclusions on disease pathogenesis is generally difficult, partially because variants often map to noncoding regions and the disease follows complex genetic traits (2, 3). Although rare, monogenic autoimmune diseases offer a unique opportunity to develop models that enable a more mechanistic understanding of the disease process, particularly if the penetrance is high (4, 5). Here, we have identified a family with IgG4-related disease (IgG4-RD) and atopic symptoms in the father and 2 daughters. Genome sequencing for all members revealed that all 3 affected members, but not the healthy mother, shared rare variants in *IKZF1*. In addition, they carried a variant in *UBR4*, suggesting that the combination of both gene variants confers high penetrance to the disease.

The Ikaros family of Kruppel zinc finger transcription factors is a gene family that has been strongly associated with an increased risk for autoimmunity. Three members, *IKZF1* (IKAROS) (rs2366293-C, rs4917014-T), *IKZF3* (AIOLOS) (rs2941509-T), and *IKZF2* (HELIOS) (rs6435760-C) have been implicated in systemic lupus erythematosus (SLE) (6, 7). There is clear evidence from GWAS studies that an *IKZF1* haplotype is associated with SLE across multiple ancestries (8). *IKZF1* haplotypes have also been found to be associated with other autoimmune diseases, such as Crohn’s disease (rs1456896) (9); ulcerative colitis (rs145689) (10), multiple sclerosis (rs2018471) (11), and type 1 diabetes mellitus (rs10272) (12). In addition to these associations, several loss-of-function (LOF) *IKZF1* variants have been described that cause immunodeficiency and autoimmunity (13). IKAROS, encoded by *IKZF1*, is a master transcription factor of lymphopoiesis (14), but the mechanisms by which it causes autoimmunity are unknown. UBR4 is an E3 ubiquitin ligase, a component of the N-degron pathway, also known as the N-end rule pathway (15, 16). N-recognizing E3 ligases bind to destabilizing N-terminal residues to induce selective proteolysis through the ubiquitin-proteasome and autophagic systems (17).

IgG4-RD is a fibroinflammatory condition characterized by a gradual and subtle progression. Organ involvement can vary but frequently includes the infiltration of the submandibular and lacrimal glands by mononuclear cells. The histological hallmarks of IgG4-RD are the frequent presence of tissue-infiltrating IgG4 plasma cells and pronounced tissue fibrosis accompanying the inflammation. Clinically, patients often have allergic manifestations along with elevated IgG4 and IgE levels. These findings have led to the hypothesis that the disease is instigated by a Th2 response (18, 19). Th2 cytokines such as IL-4 and IL-13 promote IgG4 and IgE class-switching as well as induce fibrosis (20, 21). The mechanisms underlying this Th2 bias in IgG4-RD remain unknown.

In the present study, we aimed to identify molecular pathways downstream of the gene variants in our family that could explain the pathogenesis of IgG4-RD as well as provide information on the general function of IKAROS in autoimmunity. We found that the IKAROS-p.(Arg183His) variant promoted transcription of the Src kinase *FYN* as a gain-of-function (GOF) variant, while the *UBR4* p.(Cys4179Ter) variant resulted in CD45 accrual by blocking its lysosomal degradation. Increased CD45 abundance in T cells and B cells activated FYN by dephosphorylating its inhibitory site. Hyperactivity of FYN in T cells not only augmented the T cell receptor (TCR) signaling pathway, but also accounted for increased Th2 polarization by preventing the degradation of JunB. In addition to proposing a disease model for IgG4-RD, our studies identify molecular pathways that may be relevant for other *IKFZ1*-associated autoimmune diseases. Moreover, the function of IKAROS and FYN in regulating Th2 polarization may explain the frequent atopic manifestations in IgG4-RD and be relevant for atopic diseases in general.

## Results

### IKZF1 and UBR4 variants in familial IgG4-RD.

**Figure 1:**
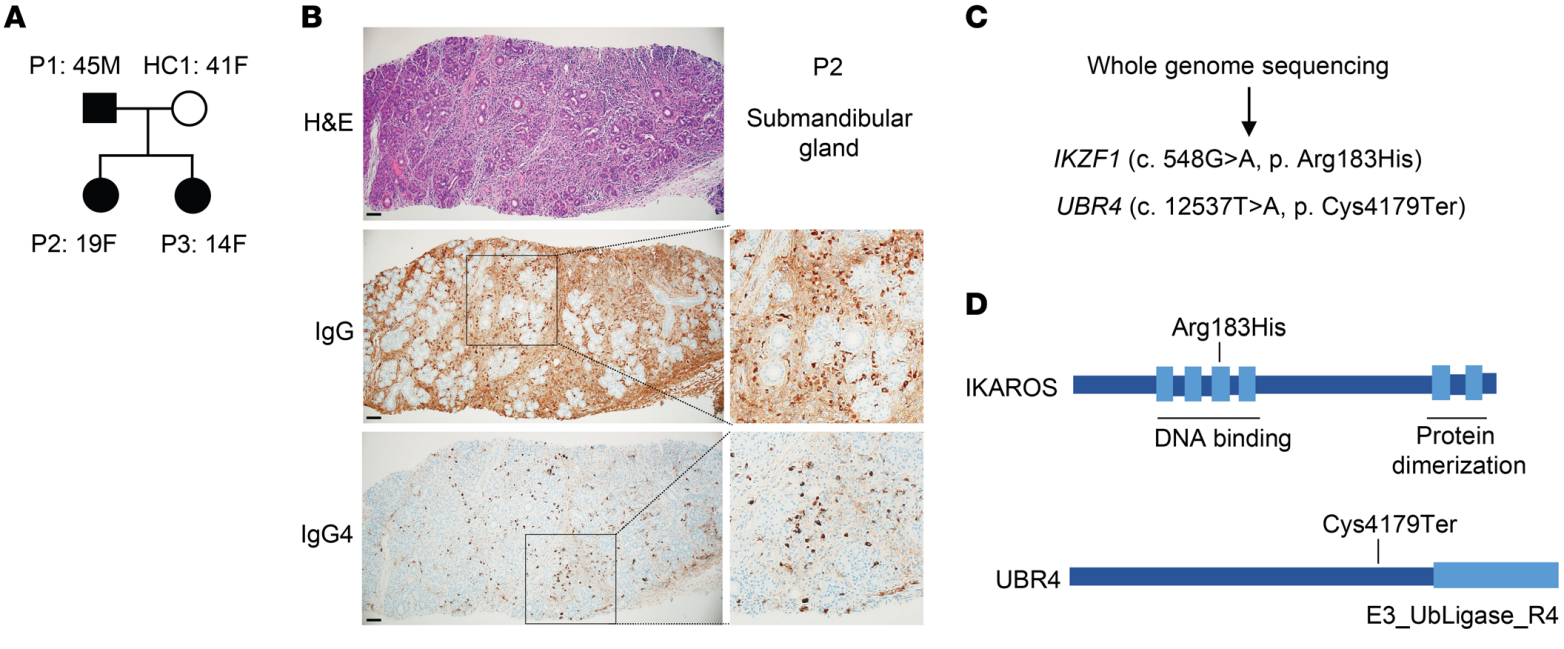
*IKZF1* and *UBR4* variants in familial IgG4-RD (**A**) Family pedigree of the patients. P2 is the index patient diagnosed with IgG4-RD. (**B**) Immunohistochemical analysis of a submandibular gland from P2 showing infiltration with IgG4^+^ cells. Scale bars: 100 μm. Original magnification, ×2 (insets). (**C**) Variants found by whole-genome sequencing that segregated with disease. (**D**) Schematic representation of variants in the IKAROS (encoded by *IKFZ1*) and UBR4 proteins.

We have identified a familial cluster, in which the father and 2 daughters have IgG4-RD and the mother is unaffected (Figure 1A). The father and older daughter presented with identical symptoms of head and neck involvement including dacryoadenitis (lacrimal gland swelling), pulmonary nodularity, bronchiectasis, adenopathy, elevated IgE and IgG4, and allergic rhinitis (Supplemental Tables 1 and 2). Histopathological analysis of a submandibular gland biopsy from the older daughter (P2) displayed infiltration with IgG4 plasma cells (Figure 1B). The younger daughter had adenopathy, markedly elevated IgE, and elevated IgG4 and allergic rhinitis but has not developed pulmonary or lacrimal gland manifestations. We performed genome sequencing for the quad (father, mother, and 2 affected daughters) and found that the father and both daughters share rare variants in *IKFZ1* (NM_006060.6:c. 548G>A, p. [Arg183His]) and in *UBR4* (NM_020765.3:c.12537T>A, p. [Cys4179Ter]) (Figure 1C and Supplemental Figure 1). The IKAROS p.(Arg183His) variant is located in the DNA-binding domain and has been identified as causing GOF (22). The *UBR4* variant in the family we studied caused an early termination and truncation of the protein, which may have compromised its function (Figure 1D). Neither variant is present in any population in the gnomAD database and both variants are absent in the unaffected mother, implying a strong likelihood of a pathogenic role of these variants either independently or in combination.

### Increased CD45 expression in affected family members.

**Figure 2:**
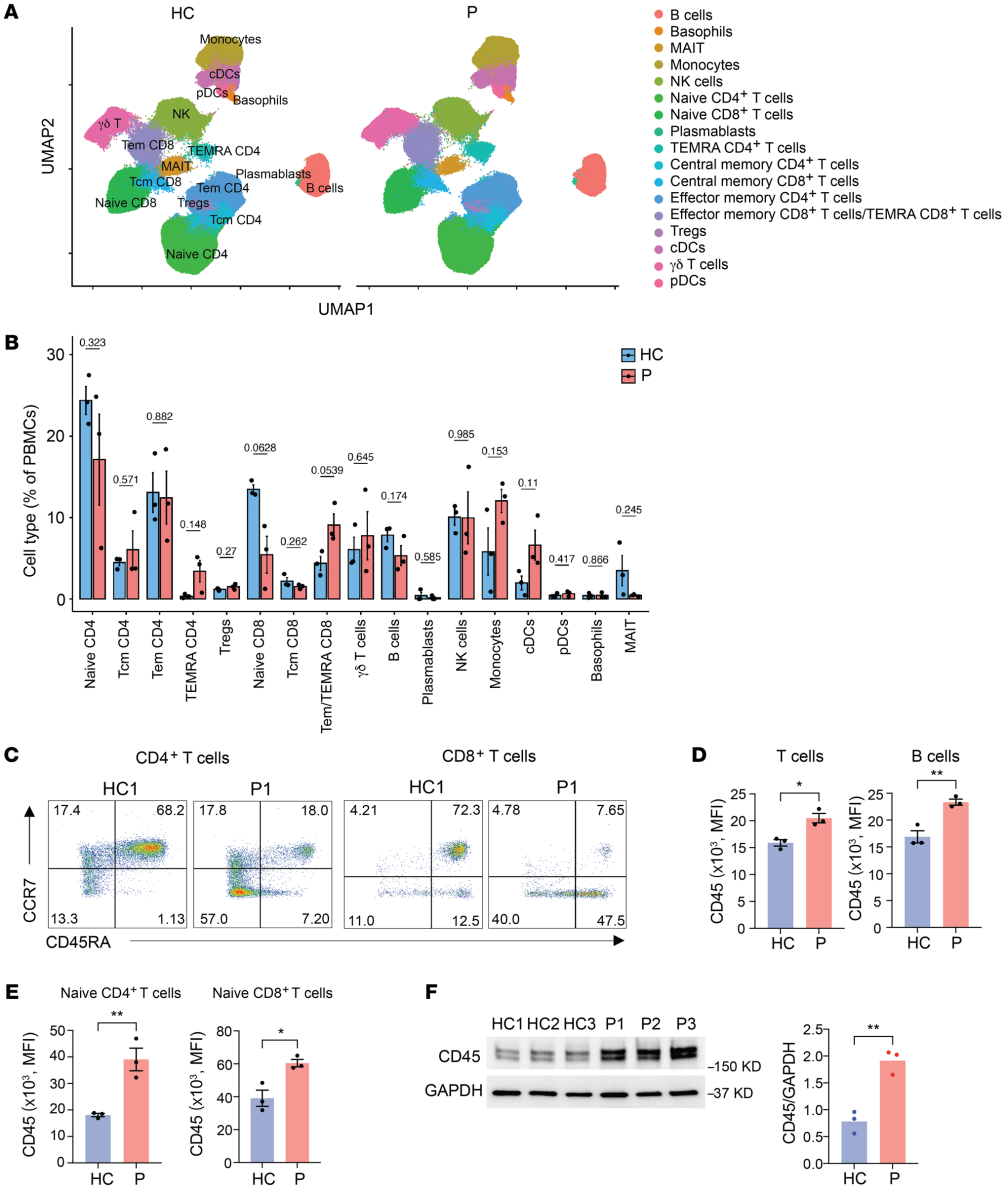
Increased cell-surface expression of CD45 in patients. (**A**) UMAP visualization of mass cytometry analysis of PBMCs from 3 healthy controls (HC) and the 3 patients (P). (**B**) Frequencies of the indicated cell types from CyTOF expressed as a percentage of PBMCs. (**C**) Flow cytometric analysis of naive and memory CD4^+^ and CD8^+^ T cell subsets as determined by CD45RA and CCR7 expression. Results are shown for age-matched HC1 and P1. (**D**) Cell-surface expression of CD45 on T cells and B cells from healthy controls (*n* = 3) and patients (*n* = 3). (**E**) Cell-surface expression of the CD45RA isoform measured by flow cytometry on naive CD4^+^ T cells and naive CD8^+^ T cells from healthy controls (*n* = 3) and patients (*n* = 3). (**F**) Immunoblot analysis of CD45 in T cell blasts derived from PBMCs from healthy controls and patients. Data are presented as the mean ± SEM. *P < 0.05 and **P < 0.01, by 2-tailed unpaired Student’s *t* test.

To determine whether the genetic variants affect the immune cell compositions, we immunophenotyped PBMCs from the 3 patients and 3 healthy adults, including the unaffected mother, using mass cytometry (cytometry by the time of flight [CyTOF], Figure 2A). Global peripheral blood population studies by CyTOF were followed by spectral cytometry focused on T cells. The uniform manifold approximation and projection (UMAP) detected the presence of regular cell populations without consistent distortions between patients and controls (Figure 2, A and B, and Supplemental Figure 2A). However, cell frequencies were highly variable, possibly due to confounding variables such as age differences between the parents and daughters or treatment in 1 daughter (Supplemental Figure 2A). We noted trends toward increased monocyte and conventional dendritic cell (cDC) frequencies and a skewing in T cell subsets from naive to effector T cells, indicating global accelerated differentiation. When comparing the aged-matched mother and father (HC1 and P1), both CD4^+^ and CD8^+^ T cells were strongly skewed toward effector memory and terminally differentiated effector memory T cells (TEMRA cells) in the patient (Figure 2C and Supplemental Figure 2, B and C). There was a trend toward downregulation of CD27, CD28, and IL-7R and upregulation of CX3CR1, which may have reflected increased effector differentiation in patients. The most impressive and consistent finding was an increased expression of CD45 on all hematopoietic cells including T and B cells irrespective of their differentiation or activation state (Figure 2, C and D). Increased expression affected all CD45 isoforms irrespective of differential splicing. CD45 expression was nearly 2-fold higher in naive T cells from patients compared with healthy controls (Figure 2E). Immunoblots of T cell blasts derived from healthy controls and patients using a pan anti-CD45 Ab confirmed the CD45 abundance (Figure 2F).

### Enhanced TCR activation in affected family members in spite of upregulated CD45 levels.

**Figure 3:**
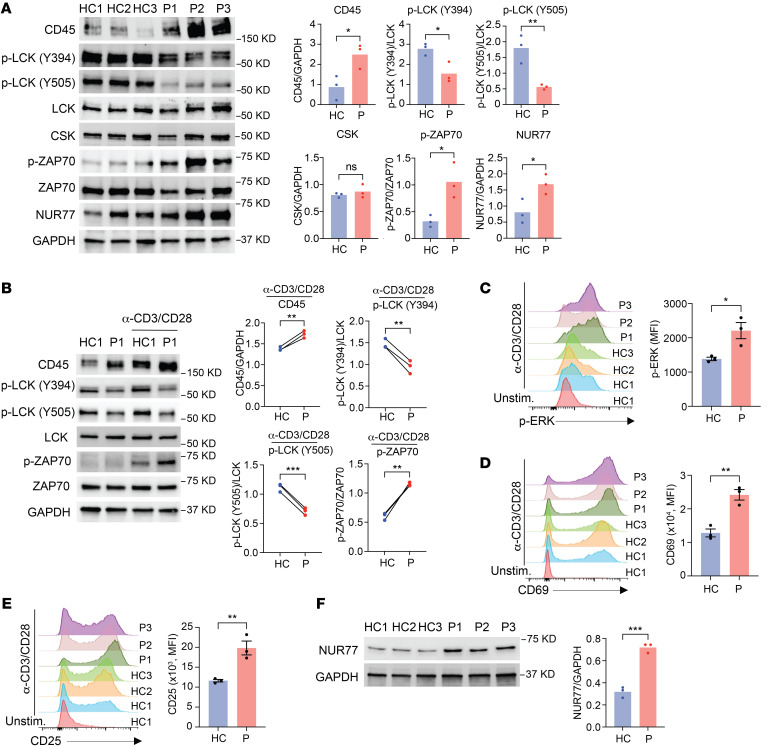
Increased constitutive and activation-induced TCR signaling in patients. (**A**) Immunoblot analysis of unstimulated PBMCs from healthy controls and patients. (**B**) Representative immunoblots of LCK and ZAP70 phosphorylation in T cell blasts derived from 1 healthy control and 1 patient (left) with or without anti-CD3/anti-CD28 (α-CD3/CD28) stimulation for 15 minutes. Summary results (right) are from 3 experiments, each with 1 healthy control and 1 patient. (**C**) p-ERK was measured by flow cytometry in T cell blasts derived from healthy controls (*n* = 3) and patients (*n* = 3) after anti-CD3/anti-CD28 stimulation for 15 minutes. (**D** and **E**) CD69 (**D**) and CD25 (**E**) expression was measured by flow cytometry on T cell blasts derived from healthy controls (*n* = 3) and patients (*n* = 3) after anti-CD3/anti-CD28 stimulation for 24 hours. Unstim., unstimulated. (**F**) Immunoblot analysis of NUR77 levels in T cell blasts derived from healthy controls and patients after anti-CD3/anti-CD28 stimulation for 24 hours. Data represent the mean ± SEM. *P < 0.05, **P < 0.01, and ***P < 0.001, by 2-tailed, unpaired (**A** and **C**–**F**) or 2-tailed, paired (**B**) Student’s *t* test.

CD45 is a membrane tyrosine phosphatase that plays essential roles in controlling TCR signaling (23). TCR signal transduction is initiated when the TCR binds to peptide-MHC complexes expressed on antigen-presenting cells (APCs), and the Src family protein tyrosine kinase LCK is recruited and phosphorylates and activates the protein tyrosine kinase ZAP70. ZAP70, in turn, phosphorylates the transmembrane adaptor protein called linker for activation of T cells (LAT). This sets the stage for the recruitment of various adaptor molecules and effector components to activate the downstream signaling cascade for T cell activation (24). Depending on the expression level, CD45 exerts positive and negative control of LCK activity by dephosphorylating the activating site at Tyr394 and the inhibitory site at Tyr505 (25–27). As expected for higher CD45 expression, we found reduced constitutive phosphorylation of LCK at both sites, which is predicted to subdue TCR signaling. Paradoxically, we found increased constitutive phosphorylation of ZAP70 in unstimulated T cells from affected family members (Figure 3A). Expression of CSK, which only phosphorylates the inhibitory tyrosine (27), was not increased. Consistent with constitutive TCR–mediated activation of T cells under homeostatic conditions (28), NUR77 was more abundant in the patients. Similarly, evidence of increased signaling was found upon stimulation. Phosphorylation of ZAP70 as well as ERK was increased after CD3/CD28 cross-linking in the patients (Figure 3, B and C) and induction of the activation markers CD69 (Figure 3D), CD25 (Figure 3E), and NUR77 (Figure 3F) was upregulated. Taken together, in spite of reduced LCK phosphorylation due to increased CD45 expression, downstream TCR signaling events and T cell activation were enhanced in the affected family members.

### Increased abundance and activity of the Src kinase FYN in affected family members.

**Figure 4:**
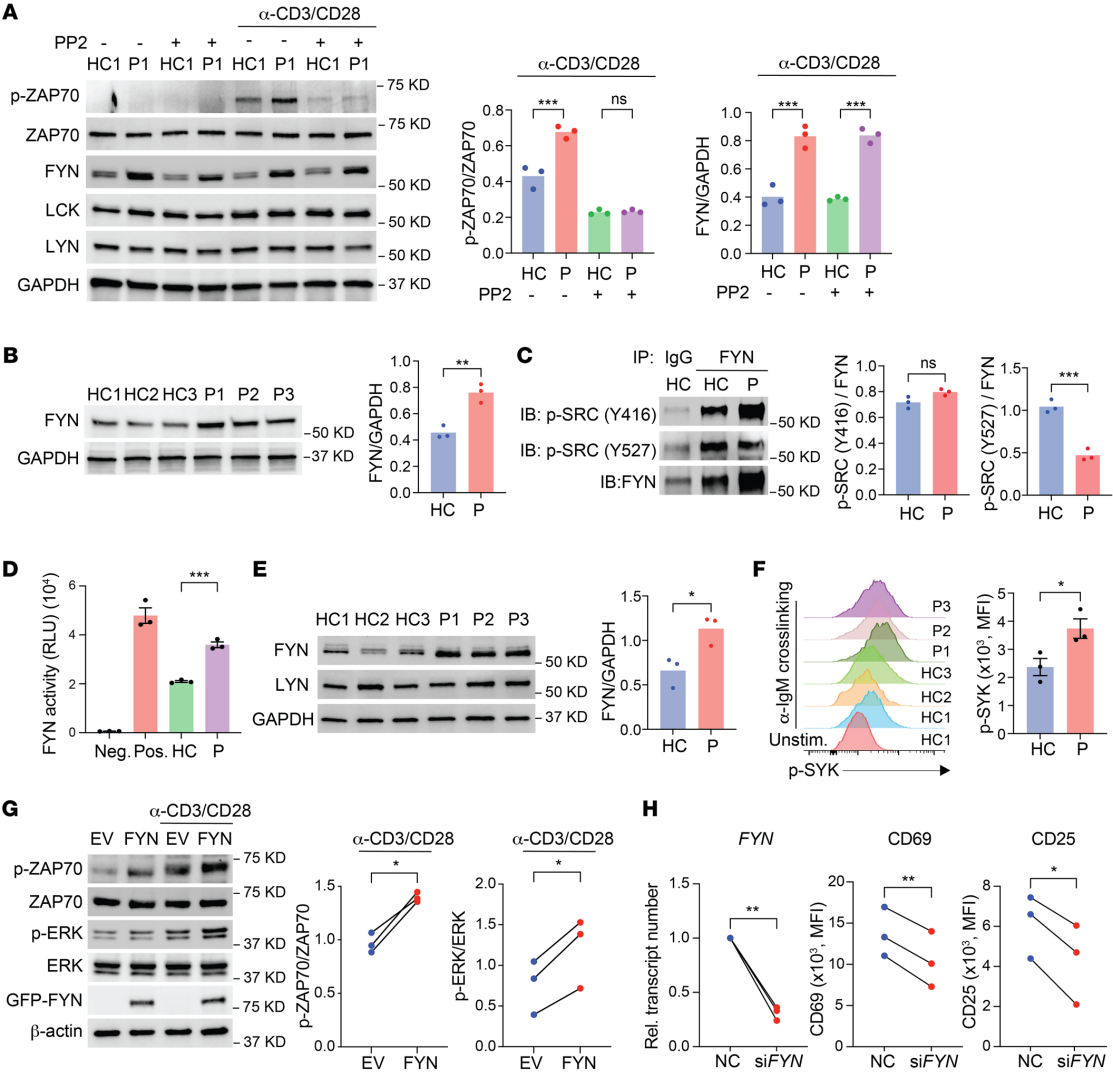
FYN abundance and activity are upregulated in the patients. (**A**) Immunoblot analysis of T cell blasts treated with or without PP2 (1 μM) left unstimulated or followed by anti-CD3/anti-CD28 stimulation for 15 minutes. Representative blot is from 1 healthy control and 1 patient and summary intensities are from 3 experiments. (**B**) Immunoblot analysis of FYN in T cell blasts derived from healthy controls (*n* = 3) and the patients (*n* = 3) after anti-CD3/anti-CD28 stimulation for 24 hours. (**C**) Immunoprecipitation of FYN from T cell blasts derived from 1 healthy control and 1 patient after 15 minutes of anti-CD3/anti-CD28 stimulation and immunoblot (IB) analysis of FYN phosphorylation at activating and inhibitory sites, normalized to FYN. (**D**) Kinase activity of FYN in T cell blasts derived from healthy controls (*n* = 3) and patients (*n* = 3) after anti-CD3/anti-CD28 stimulation for 15 minutes. Negative (Neg.) and positive (Pos.) indicate results from system controls. (**E**) Immunoblot analysis of EBV-transformed B cell lines from healthy controls (*n* = 3) and the patients (*n* = 3). (**F**) SYK phosphorylation measured by flow cytometry in EBV transformed B cell lines from healthy controls (*n* = 3) and patients (*n* = 3) after anti-IgM cross-linking for 5 minutes. (**G**) Immunoblot analysis of naive CD4^+^ T cells from healthy individuals; cells were lentivirally transduced with empty vector (EV) or a vector encoding *FYN*. Cells were left unstimulated or stimulated with anti-CD3/anti-CD28 for 15 minutes. Representative blots and intensity results from 3 independent experiments are shown. (**H**) T cell blasts derived from healthy controls (*n* = 3) and patients (*n* = 3) were transfected with control siRNA or *FYN* siRNA. *FYN* mRNA levels were determined by qPCR. CD69 and CD25 levels were determined by flow cytometry. Rel., relative. Data represent the mean ± SEM. *P < 0.05, **P < 0.01, and ***P < 0.001, by 1-way ANOVA (**A**), 2-tailed, unpaired Student’s *t* test (**B**, **D**, **E**, and **F**), or 2-tailed, paired Student’s *t* test (**G** and **H**).

To determine whether TCR signaling in patients was dependent on Src kinase activity, we treated the T cells with the Src family kinase inhibitor PP2 at intermediate concentrations and then examined TCR signaling after activation. PP2 inhibited ZAP70 phosphorylation equally in T cells from patient P1 and from a healthy control (Figure 4A). LCK is generally considered to be the main Src kinase in TCR signaling. To determine whether another Src kinase accounted for the increased TCR signaling in affected family members, we quantified the expression of FYN and LYN. FYN abundance was significantly higher in T cells from the patient, whereas the abundance of LYN and LCK did not differ (Figure 4A). Increased FYN expression in T cells was a consistent feature of all 3 patients (Figure 4B).

In contrast to LCK, which is dephosphorylated by CD45 at both activating and inhibitory tyrosine residues, CD45 only targets the inhibitory site in FYN and LYN. Increased CD45 is therefore predicted to inhibit LCK but stimulate FYN activity, as was shown for B cell antigen receptor signaling (26). Indeed, we found reduced phosphorylation only at the inhibitory FYN site Y527 in T cells from patients (Figure 4C). In contrast to LCK, we found that phosphorylation at the activating site at Y416 of FYN was increased. Also, we confirmed enhanced FYN kinase activity in patients’ T cells (Figure 4D).

As the major Src family kinase in B cells, FYN plays a critical role in B cell receptor (BCR) signaling. Since B cells from patients were too few for protein studies, we examined FYN levels in EBV-transformed B cells. Like in T cells, we found that expression of FYN, but not LYN, was significantly increased in EBV cells from patients compared with those from healthy controls (Figure 4E). We also found that increased FYN was functional, as documented by increased SYK phosphorylation after IgM cross-linking (Figure 4F).

To validate that FYN was responsible for enhanced T cell signaling and activation, we examined TCR signaling after FYN overexpression. Ectopic expression of FYN in healthy T cells resulted in increased ZAP70 and ERK phosphorylation (p-ERK) upon CD3/CD28 cross-linking (Figure 4G). Conversely, knockdown of *FYN* in T cells from the patients mitigated the T cell activation (Figure 4H). In summary, we found that FYN was more abundant in the patients, which, in the presence of increased CD45 expression, led to enhanced FYN activity, increasing TCR and BCR signaling.

### Synergistic function of the IKFZ1 and UBR4 variants in causing FYN hyperactivity.

**Figure 5:**
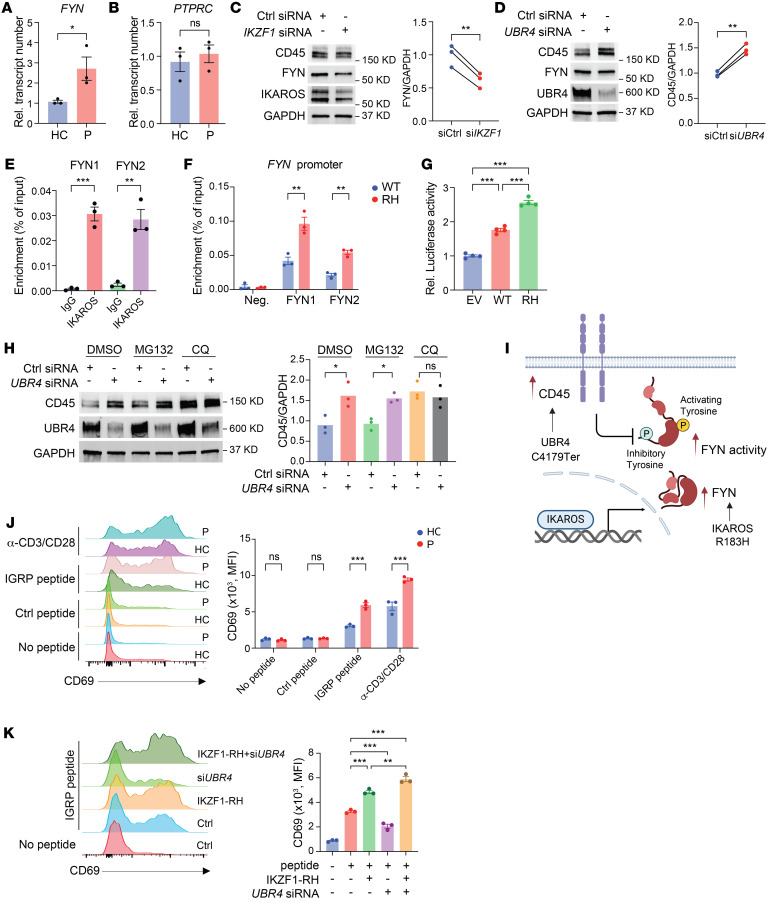
Synergistic function of IKAROS and UBR4 variants in causing FYN hyperactivity. (**A**) *FYN* expression was determined by qPCR in T cell blasts derived from healthy controls (*n* = 3) and patients (*n* = 3). (**B**) *PTPRC* expression was determined by qPCR in T cell blasts derived from healthy controls (*n* = 3) and patients (*n* = 3). (**C**) Immunoblot analysis of total T cells from healthy individuals transfected with control (Ctrl) siRNA or *IKZF1* siRNA. (**D**) Immunoblot analysis of total T cells from healthy individuals transfected with control siRNA or *UBR4* siRNA. (**E** and **F**) ChIP-qPCR analysis of IKAROS binding with 2 *FYN* promoter primer sets. Total T cells derived from healthy controls were transduced with WT IKAROS (**E**) or with WT or the R183H *IKFZ1* variant (**F**). (**G**) HEK293T cells were transfected with *FYN* promoter reporter constructs together with empty vector, vector encoding WT IKAROS, or the R183H variant (RH). Luciferase activity was determined 24 hours after transfection. (**H**) Immunoblot analysis of total T cells from healthy individuals transfected with control siRNA or *UBR4* siRNA followed by DMSO, MG132 (10 μM), or CQ (50 μM) treatment for 8 hours. (**I**) Schematic representation of the synergistic function of IKAROS and UBR4. (**J**) T cells derived from healthy controls (*n* = 3) and patients (*n* = 3) were transduced with the T1D2 TCR transgene. Cells were stimulated with no peptide, control peptide, or IGRP peptide–loaded APCs and analyzed for the expression of CD69 by flow cytometry. Anti-CD3/anti-CD28 stimulation served as a positive control. (**K**) T1D2 TCR–transgenic T cells derived from a healthy control were transduced with the R183H *IKZF1* variant or transfected with *UBR4* siRNA. CD69 expression was determined after stimulation with no peptide or IGRP peptide–loaded APCs. Data represent the mean ± SEM. *P < 0.05, **P < 0.01, and ***P < 0.001, by 2-tailed, unpaired Student’s *t* test (**A**, **B**, **E**, and **F**), 2-tailed, paired Student’s *t* test (**C** and **D**), 1-way ANOVA (**G**, **H**, and **K**), or 2-way ANOVA (**J**).

To identify the molecular mechanisms by which FYN and CD45 expression were increased in patients, we first examined transcripts of *FYN* and *PTPRC* (encoding CD45). *FYN*, but not *PTPRC*, transcripts were increased in the patients (Figure 5, A and B), indicating that FYN upregulation occurred at the transcriptional level, whereas CD45 occurred at the posttranscriptional level. To investigate the contribution of the gene variants in FYN and CD45 upregulation, we silenced *IKZF1* and *UBR4* and determined FYN and CD45 protein expression (Figure 5, C and D). Knockdown of *IKZF1* resulted in reduced FYN level but had no effect on CD45 (Figure 5C). Conversely, knockdown of *UBR4* increased CD45 but did not affect FYN expression (Figure 5D).

In further support of the function of IKAROS in the transcriptional regulation of *FYN*, we performed ChIP and found that IKAROS bound to *FYN* promoter sequences (Figure 5E and Supplemental Figure 3A). The IKAROS-R183H variant showed higher binding to the *FYN* promoter when transfected into HEK293T cells (American Type Culture Collection [ATCC], CRL-3216, Supplemental Figure 3B) or primary T cells (Figure 5F), consistent with its characterization as a GOF variant with increased DNA binding (22). As further evidence, *FYN* promoter luciferase activity in cells transfected with the IKAROS-R183H variant was increased (Figure 5G).

Since UBR4 is an E3 ubiquitin ligase controlling protein degradation, we hypothesized that the UBR4 truncation compromised its function and thereby reduced CD45 protein turnover. *UBR4* silencing in T cells increased CD45 expression. We then treated the cells with MG132 or chloroquine (CQ) to block the proteasomal and lysosomal degradation pathways, respectively. Blockade of the lysosomal, but not the proteasomal, pathway abolished the effect of *UBR4* silencing on stabilization of CD45 expression (Figure 5H). These data support the model of synergistic activity of the *IKAROS* and *UBR4* variants. The IKAROS-R183H variant promotes *FYN* expression, while the *UBR4* variant stabilizes CD45 to further increase FYN activity (Figure 5I). As predicted by this model, *UBR4* silencing alone in the absence of FYN overexpression reduced activation-induced CD69 expression (Supplemental Figure 3C).

To examine whether these variants in the patients sufficiently lower the threshold for T cell activation to break tolerance of self-reactive T cells, we utilized an experimental system involving T1D2, a low-avidity, self-reactive TCR isolated from a patient with type 1 diabetes mellitus (29–31). The T1D2 TCR recognizes a peptide derived from islet autoantigen islet-specific glucose-6 phosphatase catalytic subunit–related protein (IGRP) in the context of HLA-DRB1*04. We transduced T cell blasts from healthy controls and patients with the TCR transgenes. The T1D2 construct includes a variable region of the human islet–specific TCR (huVα and huVβ) and a constant region of murine TCR (muCα and muCβ). Flow cytometry using anti–mouse TCR β chain Ab showed similar TCR transgene expression in T cells from the individuals (Supplemental Figure 3D). Upon stimulation with HLA-DRB1*04^+^ PBMC and IGRP peptide, T cells with IKZF1 and UBR4 variants from patients exhibited increased expression of activation markers compared with transduced T cells from healthy adults (Figure 5J and Supplemental Figure 3E). To provide further support for the interpretation of synergistic activity of the variants, T1D2 TCR–transgenic T cells derived from a healthy control were transduced with the R183H *IZKF1* variant and/or transfected with *UBR4* siRNA. We measured CD69 expression after stimulation with no peptide or with IGRP peptide–loaded APCs (Figure 5K).

### FYN activity accounts for Th2 skewing in affected family members.

**Figure 6:**
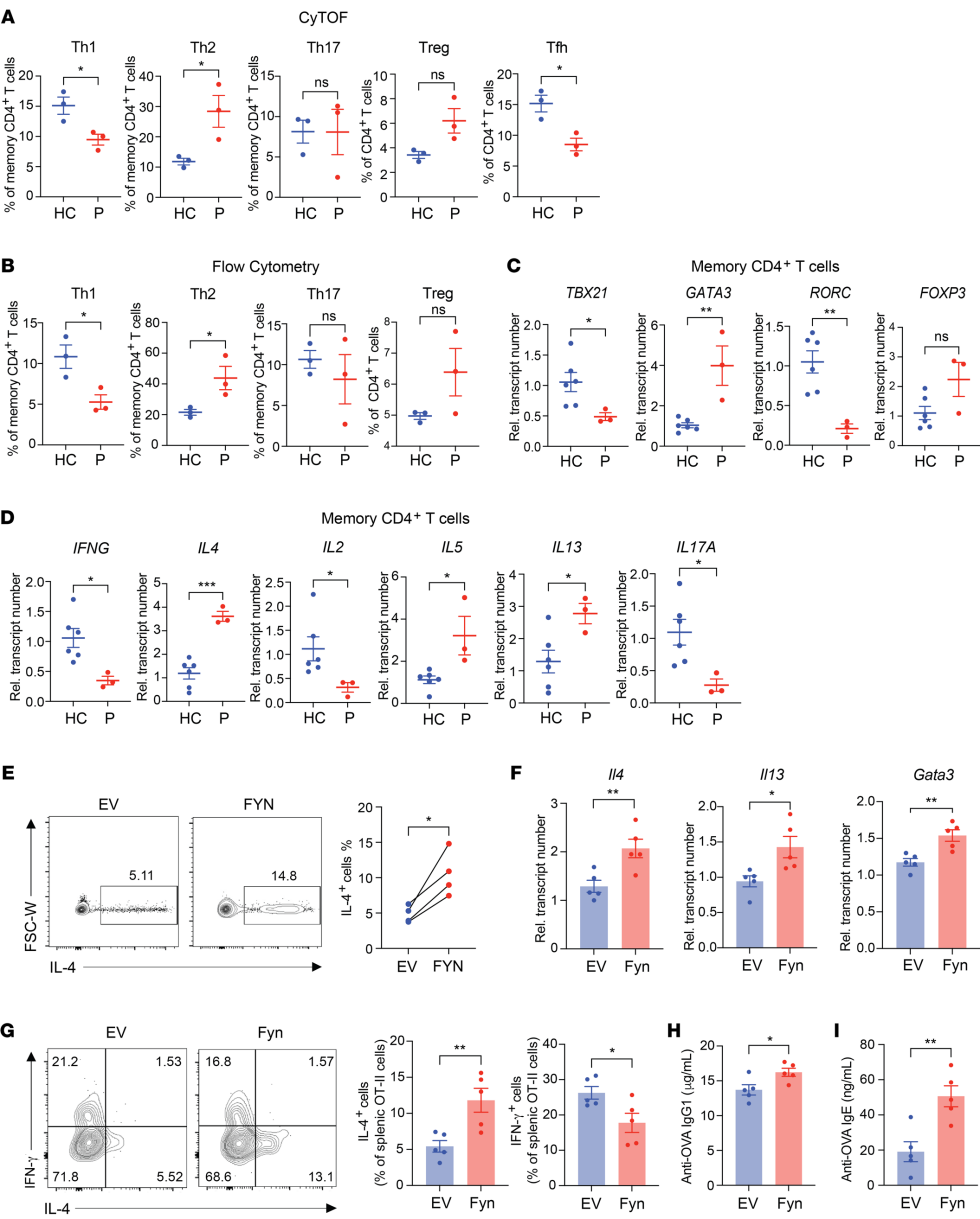
Th2 skewing in affected family members is caused by an IKAROS-mediated increase in FYN. (**A** and **B**) Frequencies of Th1 (CD3^+^CD4^+^CCR4^–^CXCR3^+^CCR6^–^), Th2 (CD3^+^CD4^+^CCR4^+^CXCR3^–^CCR6^–^), Th17 (CD3^+^CD4^+^CCR4^+^CXCR3^–^CCR6^+^), Treg (CD3^+^ CD4^+^CD25^+^CD127^–^), and T follicular helper (Tfh) (CXCR5^+^CD4^+^) cells in healthy controls (*n* = 3) and patients (*n* = 3) were determined by mass cytometry (**A**) and confirmed by flow cytometry (**B**). (**C** and **D**) Memory CD4^+^ T cells from healthy controls (*n* = 6) and patients (*n* = 3) were stimulated with PMA and ionomycin for 2 hours. Plots show the relative transcript expression of lineage-determining transcription factors (**C**) and intracellular cytokines (**D**). (**E**) Naive CD4^+^ T cells from healthy individuals were lentivirally transduced with empty vector or vector encoding *FYN*. Cells were cultured on anti-CD3/anti-CD28–coated plates for 7 days under nonpolarizing conditions. Intracellular production of IL-4 in transduced cells was determined after PMA and ionomycin treatment for 6 hours. FSC-W, forward scatter width. (**F**–**I**) Empty vector control or FYN retrovirally transduced naive OT-II CD4^+^ T cells were adoptively transferred into recipient mice followed by NP-OVA immunization. (**F**) At day 8 after immunization, mRNA expression in splenocytes was analyzed by qPCR. (**G**) Intracellular production of IFN-γ and IL-4 in transduced cells was then determined after PMA and ionomycin treatment for 6 hours. Serum anti-OVA IgG1 (**H**) and IgE (**I**) levels were determined by ELISA. Data represent the mean ± SEM. *P < 0.05, **P < 0.01, and ***P < 0.001, by 2-tailed, unpaired (**A**–**D** and **F**–**I**) or 2-tailed, paired (**E**) Student’s *t* test.

One of the hallmarks of IgG4-RD is a bias toward Th2 differentiation that may explain the high incidence of allergic manifestations. Clinically, affected family members had a history of allergy consistent with nonfamilial IgG4-RD patients. Routine laboratory work showed elevated serum levels of IgG4 and IgE (Supplemental Table 2). We analyzed the CyTOF data (Figure 2A) for the expression of chemokine receptor patterns and indeed found an increase in CCR4^+^CCR6^–^CXCR3^–^ Th2 and a decrease in CCR4^–^CCR6^–^CXCR3^+^ Th1 cell frequencies (Figure 6A). These data were confirmed by conventional flow cytometry analysis of chemokine receptor expression (Figure 6B). Moreover, we sorted CD4^+^ memory T cells and determined the transcript number of lineage-determining transcription factors by quantitative PCR (qPCR). Expression of *GATA3* was increased, while expression of *TBX21* and even more so of *RORC* (RORCγt) was low in the affected members (Figure 6C). FOXP3 expression was higher in 2 of the 3 affected family members; the difference did not reach significance, given the limited power of the sample size. We subsequently stimulated isolated CD4^+^ memory T cells with ionomycin and PMA and determined transcript numbers for cytokines. The results paralleled those of transcription factor typing. Transcription of *IL4*, *IL5*, and *IL13* was markedly increased, while *IFNG* and *IL17* transcription was reduced (Figure 6D). Interestingly, IL-2 production was also decreased, indicating loss of polyfunctionality.

To investigate whether FYN hyperactivity has a role in the development of Th2 responses, we transduced naive CD4^+^ T cells from healthy individuals with *FYN* and cultured them under nonpolarizing conditions. We found that ectopic expression of FYN promoted the generation of IL-4–producing cells after 7 days of culturing under nonpolarizing conditions (Figure 6E). *IL4* transcripts were already upregulated after 2 days, while *IFNG* and *IL17* transcripts were not affected, consistent with the interpretation that the reduced Th1 and Th17 frequencies were not a direct effect of FYN (Supplemental Figure 4A).

To validate the role of FYN in Th2 polarization in vivo, we used an OVA immunization mouse model. We retrovirally transduced naive CD4^+^ T cells from OT-II mice with *Fyn* or an empty vector control and transferred the cells to recipient mice followed by 4-hydroxy-3-nitrophenylacetyl-ovalbumin (NP-OVA) immunization (Supplemental Figure 4B). Mice that received FYN-overexpressing OT-II cells had increased transcript levels of the Th2 genes *IL4*, *IL13*, and *Gata3* (Figure 6F). FYN-overexpressing OT-II cells also showed increased Th2 differentiation, as indicated by intracellular cytokine IL-4 and IFN-γ staining (Figure 6G). Moreover, mice that received FYN OT-II cells had elevated anti–OVA IgG1, IgG2c, and IgE serum levels, whereas total IgG levels were unchanged (Figure 6, H and I, and Supplemental Figure 4, C and D). Taken together, these data suggest that elevated FYN function promoted Th2 cell differentiation in vitro and in vivo.

### The IKAROS variant drives Th2 polarization through FYN and JunB.

**Figure 7:**
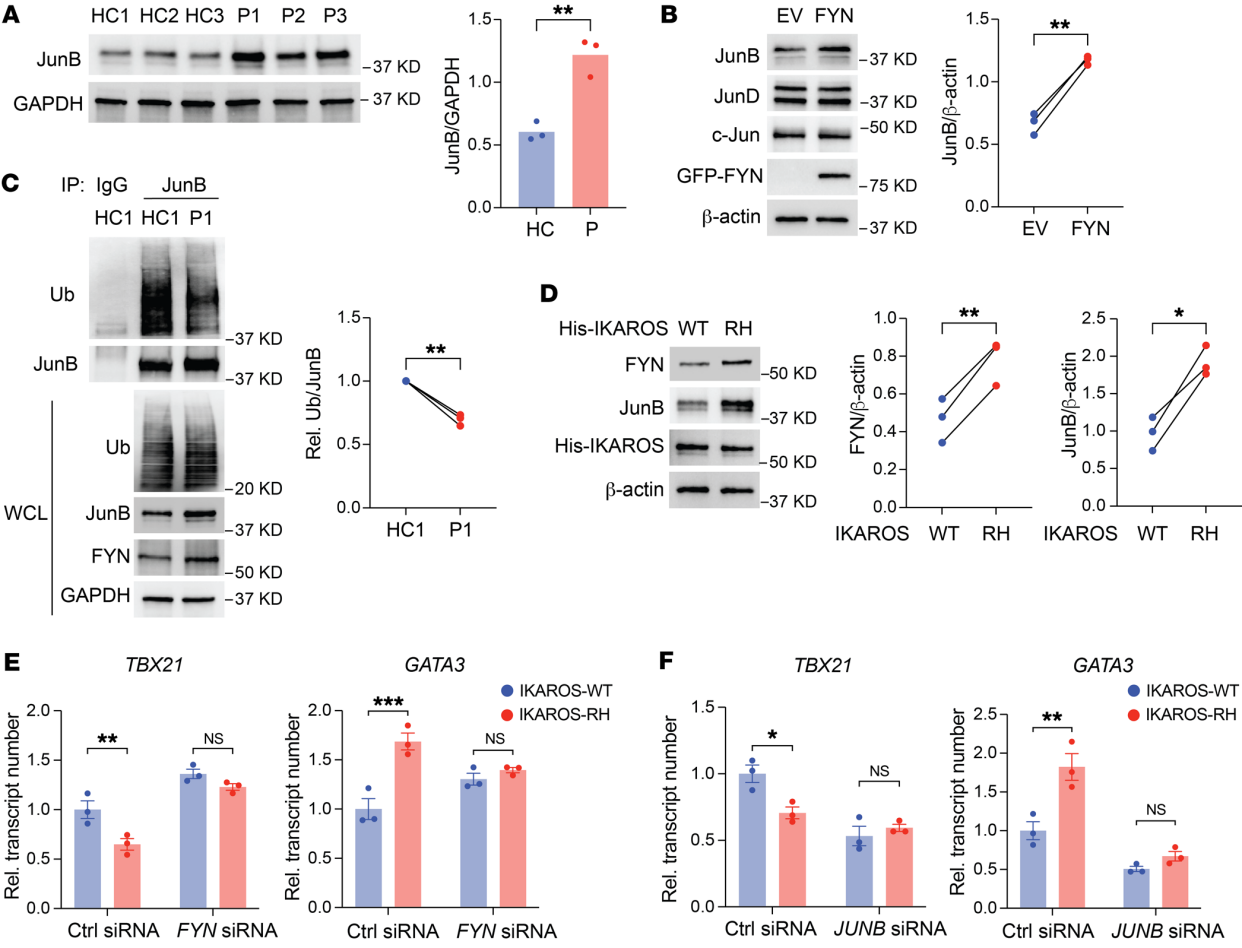
IKAROS GOF enhances Th2 polarization through the FYN/ITCH/JunB axis. (**A**) Immunoblot analysis of JunB in T cell blasts derived from healthy controls (*n* = 3) and the patients (*n* = 3) after anti-CD3/anti-CD28 stimulation for 24 hours. (**B**) Immunoblot analysis of JunB in T cells from healthy individuals; the cells were lentivirally transduced with empty vector or an *FYN*-encoding vector. JunD and c-Jun were stained as a control to rule out nonselectivity. (**C**) Immunoprecipitation and immunoblot analysis of ubiquitinated (Ub) JunB in T cell blasts derived from a healthy control and a patient after anti-CD3/anti-CD28 stimulation for 6 hours. Representative blots and summary of 3 experimental replicates are shown. (**D**) Immunoblot analysis of FYN and JunB in naive CD4^+^ T cells from healthy individuals; the cells were lentivirally transduced with WT *IKFZ1* or R183H variant–encoding vectors. (**E** and **F**) WT *IKFZ1* and R183H (RH) lentivirally transduced naive CD4^+^ T cells from healthy individuals were transfected with control or *FYN* siRNA (**E**) or control or *JUNB* siRNA (**F**). Cells were cultured on anti-CD3/anti-CD28–coated plates for 7 days. mRNA expression of *TBX21* and *GATA3* was determined by qPCR after PMA and ionomycin treatment for 2 hours. Data represent the mean ± SEM (**E** and **F**). *P < 0.05, **P < 0.01, and ***P < 0.001, by 2-tailed, unpaired Student’s *t* test (**A**), 2-tailed, paired Student’s *t* test (**B**–**D**), or 2-way ANOVA (**E** and **F**).

Next, we sought to determine the mechanisms by which FYN promotes Th2 differentiation. It has been reported that the E3 ubiquitin ligase ITCH is modulated by FYN. Tyrosine phosphorylation of ITCH by FYN reduces its interaction with its substrate JunB (32). Thus, FYN hyperactivity may result in JunB stabilization. Of note, JunB has been identified as a transcription factor controlling Th2 differentiation by regulating IL-4 expression (33–35). To test the hypothesis that the IKAROS variant promotes Th2 responses by stabilizing JunB, we first determined that JunB levels were increased in T cells from the affected family members (Figure 7A). Forced expression of FYN in T cells from healthy adults elevated JunB levels without affecting the expression of JunD or c-Jun, consistent with the model showing that the IKAROS variant functions through FYN (Figure 7B). UBR4 silencing reduced JunB expression, likely reflecting reduced T cell activation (Supplemental Figure 5). To confirm that the mechanism involves JunB stabilization, we performed ubiquitination assays and found reduced ubiquitination of JunB in the affected father (Figure 7C). These results indicate that increased FYN activity in the patients led to reduced ubiquitination and stabilization of JunB.

To further corroborate that the IKAROS GOF variant affects JunB expression through the regulation of FYN expression, we examined FYN and JunB levels in T cells expressing WT IKAROS or IKAROS-R183H. We found that the IKAROS-R183H variant resulted in increased FYN and JunB abundance (Figure 7D), along with increased transcript levels of the Th2 lineage transcription factor *GATA3* but decreased expression of the Th1 transcription factor *TBX21* (Figure 7, E and F). Silencing *FYN* (Figure 7E) or *JUNB* (Figure 7F) in WT IKAROS did not show the predicted lineage-specific effects, transcript numbers of both *TBX21* and *GATA3* increased or decreased in parallel, indicating global effects of forced IKAROS overexpression on T cell activation. In contrast, when we compared WT IKAROS and IKAROS-R183H–overexpressing T cells, we found that silencing of *FYN* or *JUNB* abrogated the difference, suggesting that the IKAROS variant promoted Th2 differentiation via FYN and JunB.

## Discussion

Here, we report the mechanisms of how *IKZF1* and *UBR4* variants in a case of familial IgG4-RD cause autoimmunity with a dominant bias for Th2 polarization. Our data provide a human genetic model of IgG4-RD that includes dysregulated Th2 polarization and is therefore pertinent at least for the large subset of patients who have concurrent allergic disease (36, 37). Equally important, we describe a setting in which 2 genetic variants cooperated to cause sustained signalosome activation downstream of antigen recognition. A central component of many autoimmune models is dysregulated TCR signaling that causes the activation of mildly self-reactive T cells and their expansion and skewing toward an inflammatory effector type. The leading example for such a genetic predisposition in human disease is the LOF variant of the protein tyrosine phosphatase non-receptor 22 (*PTPN22*), which is highly associated with several autoimmune diseases including SLE, autoimmune diabetes mellitus, and rheumatoid arthritis (38). PTPN22 functions to dephosphorylate key activating tyrosine residues within LCK and ZAP70 in the TCR signaling pathway in concert with its binding partner, C-terminal Src kinase (CSK) (39–41). In the case of our familial cluster, a related pathway involving decreased degradation of CD45 resulted in unopposed FYN activation that amplified the signalosome downstream of the antigen receptor in T and B cells and was involved in Th2 polarization. Finally, our functional insights into the GOF variant of IKAROS in mature T cell responses is relevant for the many autoimmune diseases associated with *IKZF1*.

IgG4-RD is considered a single entity mainly on the basis of the histological features of lymphoplasmacytic infiltrates with dominant IgG4-producing plasma cells and a characteristic pattern of tissue fibrosis, although the clinical presentations and organ involvements are very disparate. Affected members in this IgG4-RD familial cluster shared the involvement of salivary glands without other organ manifestations such as autoimmune pancreatitis, retroperitoneal fibrosis, or chronic periaortitis. Elevated IgG4 and frequently elevated IgE serum concentrations, both isotype switches driven in humans by IL-4 (42), have initially led to the concept that IgG4-RD is an autoimmune Th2 disease (43). Subsequent studies have confirmed that the prevalence of clinical or laboratory evidence for atopic disease is high in IgG4-RD, but a large subset of patients lacked atopic symptoms, suggesting that an aberrant Th2 response may be an epiphenomenon of IgG4-RD (36, 37). Our mechanistic studies in a genetic model of the disease document that the increased induction of a Th2 response is an integral feature of the disease. The GOF variant of IKAROS causes increased transcription of *FYN* in T cells that favors the induction of a Th2 response through stabilization of JunB. These findings suggest that the disease is heterogeneous, with a Th2 response being a critical element in a large subset of patients with IgG4-RD. This concept is supported by model systems of IgG4-RD beyond this familial cluster. Hoshino et al. published a study of 8 individuals with inflammatory, autoimmune, and allergic symptoms, who shared the heterozygosity for the IKAROS variants [p.(Arg183Cys/His)] identical or similar to those described in our study’s familial cluster. The clinical presentations of these 8 individuals were more variable than ours, however, they also shared atopic features in 4 of the 8 patients with IgG4-RD (22). Functional studies, both in our study and the other publication (22), showed typical cellular hallmarks such as T and B cell activation, Th2 differentiation, and plasma cell proliferation. Also, a recently reported mouse model resembling features of IgG4-RD directly linked a Th2 bias to disease. In this model, a LOF variant of the TCR signalosome adaptor molecule LAT resulted in sustained activation of autoreactive, low-avidity T cells with Th2 polarization. In summary, in these genetic models of IgG4-RD, a Th2 bias is an integral component of the disease.

In most disease models, T cell–mediated autoimmune manifestations require persistent triggers of T cell activation. As discussed above, the LOF *PTPN22* variant is an example. In the LAT dysfunction model, the sustained activation is provided by CD28 signaling. In the case of the family in our study, this signal is coming from a variant of the E3 ubiquitin ligase UBR4 that causes an early termination of UBR4 protein translation before its ubiquitin ligase domain. The LOF of UBR4 interferes with CD45 protein turnover through the lysosomal pathway. In turn, increased expression of CD45 has a positive regulatory role for FYN, which is upregulated by the IKAROS variant. Ubiquitination, a posttranslational protein modification, serves as a regulatory mechanism for a wide array of cellular processes. It influences intracellular signaling and guides protein degradation, either through the proteasome pathway or the autophagy-lysosome system (44). Although UBR4 has so far not been implicated, ubiquitination has been identified as a critical component in the development of autoimmune disease. Somatic variants in *UBA1*, the major E1 enzyme that initiates ubiquitination, result in systemic autoinflammation (45). Several E3 ubiquitin ligases have also been shown to play important roles in autoimmunity (46, 47).

IKAROS is a master regulator of lymphopoiesis. Mice homozygous for an *IKZF1*-null variant fail to develop T cells (14, 48, 49). It is generally accepted that it plays a major role in controlling autoimmunity, but its function in regulating T cell responses is complex, and a better understanding is needed to define its contribution to autoimmunity. This is the more important, since therapeutic interventions to degrade IKAROS are becoming available. Lenalidomide and iberdomide induce degradation of IKAROS and AIOLOS through the cereblon ubiquitin ligase (50, 51).

Germline *IKZF1* single-nucleotide variants and deletions have been linked to primary immunodeficiencies. Most of these variants are LOF presenting as haploinsufficiency, defective dimerization, or dominant-negative function (52–58). In addition to a higher risk of bacterial infections and malignancies, these variants are frequently associated with autoimmune manifestations. The mechanisms by which these LOF variants cause immune dysregulation remain largely undetermined.

GWAS have found that an *IKZF1* risk haplotype confers an increased susceptibility to develop SLE. Sharabi et al. proposed that reduced activity of IKAROS accounts for its function in SLE pathogenesis (59). Forced overexpression of IKAROS leads to reduced transcription of the protein phosphatase PP2. Conversely, silencing of IKAROS enhances the expression of PP2A, suggesting that IKAROS acts as a repressor of PP2A expression. PP2A levels are increased in patients with SLE. T cell defects in SLE including decreased IL-2 and increased IL-17 production as well as decreased CD3ζ and increased FcRγ and CREMα expression have been attributed to PP2A activity (59). Likewise, we have found that reduced IKAROS expression in T cells from patients with rheumatoid arthritis results in increased activity of the arachidonic acid–regulated calcium ARC channel due to increased expression of ORAI3 and T cell activation in the synovial environment (60). Also, in mouse models, IKAROS prevents autoimmunity by promoting BCR anergy and restraining TLR signaling, consistent with the notion that IKAROS deficiency breaks tolerance (61).

In contrast to this concept of IKAROS deficiency causing autoimmunity, a recent treatment trial with iberdomide, a cereblon modulator promoting degradation of IKAROS, showed benefit in patients with SLE (6). The *IKZF1* variant linked to disease in our IgG4-RD familial cluster is a GOF variant and therefore provides a unique opportunity to mechanistically define biomarkers that may correlate with responsiveness to IKAROS depletion. We identified transcriptional upregulation of *FYN* as a hallmark feature of IKAROS GOF. FYN expression in T cells modified TCR signaling important for self/non-self discrimination. Equally important, FYN expression was biased toward a Th2 response by interfering with JunB degradation (32, 33). A beneficial effect of IKAROS depletion is therefore more likely in patients with autoimmunity who also have evidence of atopic disease. This is clearly the case for IgG4-RD, in which allergic and atopic symptoms are common (62, 63). Also, the frequency of allergic rhinitis, allergic conjunctivitis, atopic dermatitis, and asthma is increased in SLE, suggesting a relationship between autoimmunity and Th2 immunity in a subset of patients with this disease (64, 65). This subset of patients with SLE may benefit from IKAROS depletion.

### Limitations.

Our studies were based on a single family with 3 affected family members. Although the mechanistic pathways were confirmed by genetic manipulation of human or mouse cells and were therefore robust, a broader conclusion regarding this disease merits caution. Also, the number of patients was small, thus limiting the power of our studies, in particular since confounding variables such as age, disease activity, and treatment may have contributed to variability in immunological phenotypes between patients. We focused on candidate pathways that we found to be abnormal in our IgG4-RD family and that were related to the identified gene variants but have likely overlooked differences that did not reach significance.

Given the central role of the IKAROS/FYN axis in Th2 polarization, it is tempting to speculate that IKAROS has a role in atopic diseases such as atopic dermatitis and that IKAROS depletion could also be beneficial (13). UBR4 is a component of the N-degron pathway, also known as the N-end rule pathway (15, 16), and is considered to have an effect on the stability of several proteins. Among its targets are signaling molecules as well as inflammatory fragments produced in the context of inflammasome activation (66, 67). Deficiency of the N-degron pathway may therefore be relevant for the regulation of an inflammatory response and a breach in tolerance through mechanisms other than CD45 overexpression, and an unbiased assessment of the proteome could be insightful.

## Methods

### Sex as a biological variable.

Affected family members with genetic variants included the father and 2 daughters. Similar findings were obtained for both sexes.

### Study design.

The aim of this study was to investigate the cellular and molecular mechanisms of *IKZF1* and *UBR4* variants in causing disease in familial IgG4-RD. For this objective, we performed phenotyping, functional, and mechanistic analyses of PBMCs from the patients compared with PBMCs from the unaffected mother and other healthy donors. Gene silencing and forced overexpression of candidate molecules was applied to confirm the role of these variants in disease-relevant pathways using in vitro assays on human cells as well as in an immune response in mice in vivo. Finally, we constructed a model of distorted T cell activation and T cell differentiation due to the variants’ functions.

### Genome sequencing.

Genome sequencing for the 4 members of the family was performed at a CLIA-certified (Clinical Laboratory Improvement Amendments of 1988) laboratory. In summary, genomic DNA was sequenced with pair-end reads on an Illumina platform. Average mean sequencing coverage was at least 40× across the genome, with a minimum threshold of 30× for any individual sample. Bidirectional sequence reads were assembled and aligned to reference sequences based on NCBI RefSeq transcripts and the human genome build GRCh37/UCSC hg19 (https://www.ncbi.nlm.nih.gov/datasets/genome/GCF_000001405.25/). The sequence data were processed through a Mayo Clinic internal pipeline, and the data were analyzed using the proprietary Illumina platform Emedgene for variant prioritization.

### Sample preparation and cell culturing.

Peripheral blood from each member of the family was collected. Control PBMC samples were obtained from leukocyte reduction system (LRS) cones from deidentified healthy donors through the Mayo Clinic Blood Bank.

PBMCs were Ficoll-isolated from peripheral blood or LRS cones. Human total T cells were isolated by negative selection with the EasySep Human T cell Isolation Kit (STEMCELL Technologies, no. 17951). Human naive CD4^+^ T cells were isolated by negative selection with the EasySep Human naive CD4 T Cell Isolation Kit (STEMCELL Technologies, no. 19555).

T cell blasts derived from patients and healthy controls were generated and maintained by stimulating PBMCs with T cell TransAct (Miltenyi Biotec, no. 130-128-758) in TexMACS Medium (Miltenyi Biotec, no. 130-097-196) supplemented with 20 IU/mL recombinant human IL-2 (Peprotech no. 200-02), according to the manufacturer’s instructions. EBV-B cells were generated by infecting PBMCs with EBV (ATCC VR-1492) in the presence of 1 μg/mL cyclosporin A (MilliporeSigma, no. 30024).

### CyTOF.

CyTOF assays were performed according to the manufacturer’s instructions for the Maxpar Human Immune Monitoring Panel Kit (Fluidigm, no. 201324). Briefly, PBMCs from healthy controls and patients were stained with Cell-IDTM Cisplatin (Fluidigm). The cells were then FcR blocked and stained with metal-tagged surface Abs (Supplemental Table 3) in Maxpar Cell Staining Buffer (Fluidigm). Cells were fixed with 1.6% formaldehyde and resuspended in Cell-ID intercalator-Ir solution (Fluidigm). Samples were analyzed on a Fluidigm Helios mass cytometry instrument. Data were collected as flow cytometry standard (FCS) files for further analysis using the Fluidigm CyTOF software (version 6.7.1014).

For UMAP visualization, a total of 100,000 cells for each sample were randomly selected and combined. Data sets from pooled cells were normalized by centralized log ratio transformation with highly variable features determined using the vst method. We performed principal component analysis (PCA) to reduce dimensionality and generated UMAPs utilizing the top 25 principal components. To identify clusters, we applied shared nearest neighbors coupled with a smart local moving algorithm at a resolution of 1.2. The analytic procedures were executed using Seurat, version 4.2.1 (68).

### Flow cytometry.

For cell-surface staining, cells were incubated with fluorescence-conjugated Abs (Supplemental Table 3) and human FcX Fc receptor blocking solution (BioLegend, no. 422301) in PBS supplemented with 2% FBS at 4°C for 30 minutes. For intracellular cytokine staining, cells were stimulated with 50 ng/mL PMA (MilliporeSigma, no. P1585) and 1 μg/mL Ionomycin (MilliporeSigma, no. I3909) in the presence of GolgiPlug (BD Biosciences, no. 555029) for 6 hours, and then fixed with Cytofix fixation buffer (BD Biosciences, no. 554655), permeabilized with 0.5% saponin (Thermo Fisher Scientific, no. J63209-AK), and stained with fluorescence-conjugated Abs (Supplemental Table 4) at room temperature for 60 minutes. For phospho-flow, T cells were stimulated with 10 μg/mL anti-CD3 (BioLegend, no. 300465) and 2 μg/mL anti-CD28 (BioLegend, no. 302943) for 15 minutes, and B cells were stimulated by cross-linking of F(ab′)2 fragments of anti-IgM (Jackson ImmunoResearch, no. 109-006-129) for 5 minutes. Cells were then fixed with Cytofix fixation buffer (BD Biosciences, no. 554655), permeabilized with methanol, and stained with fluorescence-conjugated Abs (Supplemental Table 4) at room temperature for 60 minutes. Dead cells were excluded from the analysis using the LIVE/DEAD Fixable Aqua Dead Cell Stain kit (Invitrogen, Thermo Fisher Scientific, no. L34957). Cells were analyzed on an LSR Fortessa (BD Biosciences). Flow cytometric data were analyzed using FlowJo software version 10 (Tree Star). Gating strategies are shown in Supplemental Figure 6.

### Immunoblot.

Cells were lysed with Pierce IP lysis buffer (Thermo Fisher Scientific, no. 87787) supplemented with protease (Roche, no. 11836170001) and phosphatase (Roche, no. 4906845001) inhibitors. Proteins were separated on denaturing 4%–15% (15-well) SDS–polyacrylamide gels (Bio-Rad, no. 4561086), transferred onto PVDF membranes (Bio-Rad, no. 1704270), and probed with the indicated Abs (Supplemental Table 4). Chemiluminescent signals were developed with SuperSignal West Femto Maximum Sensitivity Substrate (Thermo Fisher Scientific, no. PI34095). Quantification of protein levels in immunoblot analysis was measured using ImageJ software (NIH).

### FYN kinase activity assay.

FYN kinase activity was measured using FYN A Kinase Enzyme System (Promega, no. V3571) together with an ADP-Glo kinase assay (Promega, no. V6930). Briefly, T cells from healthy controls and patients were activated by anti-CD3/anti-CD28 Ab for 15 minutes. Cell lysates were prepared and incubated overnight with anti-FYN Ab (Abcam, no. 1881) plus protein A/G beads (Thermo Fisher Scientific, no. 88802). FYN pull-downs were incubated with substrate/ATP in kinase buffer for 1 hour at room temperature. The ATP remaining after completion of the kinase reaction was depleted prior to an ADP-to-ATP conversion step; newly synthesized ATP was quantified by ADP-Glo kinase assay using luciferase reactions. Reactions without FYN protein or with full-length recombinant human FYN protein were used as negative and positive controls, respectively.

### RNA extraction and qPCR.

Total RNA was isolated with the RNeasy Plus Mini kit (QIAGEN, no. 74134) and was reverse transcribed using the iScript cDNA Synthesis kit (Bio-Rad, no. 1708891). qPCR was performed on a QuantStudio 6 system (Applied Biosystems) using PowerUp SYBR Green Master Mix (Applied Biosystems, no. A25742) according to the manufacturer’s instructions. Gene expression was normalized to the internal control *RPL13A* or to *Gadph* transcripts. The primer sets used in this study are shown in Supplemental Table 5.

### Transfection and lentiviral transduction.

T cells were transfected with ON-TARGETplus nontargeting siRNA pool, SMARTpool *IKZF1* siRNA, SMARTpool *UBR4* siRNA, or SMARTpool *JunB* siRNA (Horizon Discovery) using the Amaxa Nucleofector system and the P3 primary cell Nucleofector kit (Lonza, no. V4XP-3024). After transfection, cells were rested for 2 hours before being activated by plate-bound anti-CD3/anti-CD28 Ab for 3 days.

For lentiviral transduction, T cells were infected by lentiviruses expressing empty vector control, *FYN*, WT *IKZF1*, or the *IKZF1* *R183H* variant in the presence of 8 μg/mL polybrene (MilliporeSigma, TR-1003). After 18 hours, cells were activated with plate-bound anti-CD3/anti-CD28 Ab for 5 days.

### ChIP-qPCR.

ChIP assays were performed using the ChIP-IT Chromatin Immunoprecipitation kit (Active Motif, no. 53009) according to the manufacturer’s instruction. Briefly, total T cells from healthy controls were transduced or HEK293T cells were transfected with WT *IKZF1* or the *IKZF1* *R183H* variant. After 24 hours, cells were fixed with fixation buffer (containing 1% formaldehyde). DNA was then sheared into small uniform fragments, and the DNA/protein complexes were immunoprecipitated using anti-IgG control Ab (Cell Signaling Technology, no. 3900) or anti-IKAROS Ab (Cell Signaling Technology, no. 9034). Immunoprecipitated DNA was purified and subjected to qPCR using the following primers *FYN*1 (5**′**-CCCTATCAAAGAGCCCCTTG-3′, 5′-AACACACAGACCTCCTCCTC-3′) and *FYN*2 (5′-CTCGAGACAGGGCTATCTTG-3′, 5′-GTTTCAGGACAGGATTCTAC-3′) or the negative control (5′-AACCTGCAAAACATGGTTATTT, 5′-AATTTGCCCAAACAGCAAGT-3′).

### Luciferase reporter assay.

HEK293T cells were transfected with the empty vector control, WT *IKZF1*, or the *IKZF1* *R183H* variant, together with the *FYN* promoter reporter clone (GeneCopoeia, no. HPRM53014-LvPG04). After 48 hours, luciferase activity was determined using the Secrete-Pair TM Dual Luminescence Assay Kit (GeneCopoeia, no. LF031) according to the manufacturer’s instructions. *Gaussia* luciferase activity for *FYN* promoter activity was normalized to the secreted alkaline phosphatase activity as the internal control of the luminescence assay.

### Peptide stimulation of TCR-transgenic autoreactive T cells.

The low-avidity islet antigen–specific TCR T1D2 utilized in this study has been previously described (29–31). T1D2 TCR is HLA-DRB1*04:01 restricted and recognizes the 20 mer peptide QLYHFLQIPTHEEHLFYVLS (IGRP- p39 position 305–324). A noncognate peptide (KWCANPDWIHIDTTPFAGLV) was used as a negative control. T cell blasts from healthy controls and affected family members were infected with lentivirus expressing T1D2 TCR in the presence of 8 mg/mL polybrene (MilliporeSigma, TR-1003). After 18 hours, cells were activated with plate-bound anti-CD3/anti-CD28 Abs for 5 days and rested for 24 hours before peptide stimulation. For stimulation of T cells expressing T1D2 TCR, PBMCs from an allogeneic HLA-DRB1*04:01 donor were used as APCs. APCs were mixed with peptide diluted in complete media (final peptide concentration 50 ng/mL) for 2 hours. Cocultures were incubated in 96-well, U-bottomed plates with 200,000 APCs and 50,000 T cells per well for 2 days in complete media without exogenous cytokines.

### Mice, adoptive transfer and NP-OVA immunization.

Naive CD4^+^ T cells specific to the chicken OVA 323–339 peptide obtained from 5-week-old OT-II TCR-transgenic mice (The Jackson Laboratory) were activated in plates coated with 8 μg/mL anti-CD3 (Invitrogen, Thermo Fisher Scientific, no. 16-0033-85) and anti-CD28 (Invitrogen, Thermo Fisher Scientific, no. 16-0281-85) Abs. Retroviruses expressing either the pMIG-II empty vector control or pMIG-II-FYN were generated using the Plat-E Retroviral Packaging Cell Line (Cell Biolabs). T cells were transduced on days 1 and 2 after activation. On day 6 after activation, retrovirally transduced GFP^+^ OT-II cells were sorted, and 1 × 10^5^ transduced cells were i.v. transferred into 6-week-old female C57BL/6 mice (The Jackson Laboratory). One day after transfer, the mice were immunized with 100 μg NP-OVA (Biosearch Technologies, no. N-5051) precipitated in 100 μL 5% alum (aluminum potassium sulfate, MilliporeSigma, no. A-6435). On day 8 after immunization, spleens and blood were collected and analyzed. Mice were housed at a temperature of 23°C ± 2°C, relative humidity of 30%–40%, and a 12-hour light/12-hour dark cycle.

### ELISA.

OVA-specific IgG1 and IgE in serum were measured using the anti–OVA IgG1 (mouse) ELISA kit (Cayman Chemical, no. 500830) and the anti–OVA IgE (mouse) ELISA kit (Cayman Chemical, no. 500840) respectively. Total IgG and OVA-specific IgG2c were measured using the mouse total IgG Ab detection kit (Chondrex, no. 3023) and the mouse anti–OVA IgG2c Ab assay kit (Chondrex, no. 3029), respectively.

### Statistics.

Statistical analysis was performed using GraphPad Prism 10 (GraphPad Software). A 2-tailed paired or unpaired Student’s *t* test or 2-way ANOVA was used for 2-group comparisons. A 1-way ANOVA with Tukey’s post hoc test was used for multigroup comparisons. A *P* value of less than 0.05 was considered statistically significant. Statistical details and significance levels can be found in the figure legends.

### Study approval.

The human studies were approved by Mayo Clinic (Rochester, Minnesota) IRBs, and all participants provided written informed consent. The animal protocols were approved by the Mayo Clinic IACUC.

### Data availability.

All data used in the figures are available in the Supporting Data Values file.

## Author contributions

QL, CMW, and JJG designed the research and interpreted data. QL, YZ, IS, QY, and HZ performed the experiments. AJ performed bioinformatics analysis. MO, KJW, and MJK identified the patients and provided clinical data. FPEV analyzed the genome-sequencing data. KC and JHB provided reagents and assisted in antigen-specific T cell experiments. QL and JJG wrote the manuscript.

## Supplementary Material

Supplemental data

Supporting data values

## Version 1 06/17/2024

In-Press Preview

## Version 2 08/15/2024

Electronic publication
