# Supplementary material for: IKZF1 and UBR4 gene variants drive autoimmunity and Th2 polarization in IgG4-related disease
Source: J Clin Invest. 2024 Jun 13;134(16):e178692. doi: 10.1172/JCI178692 (PMC11324302; doi:10.1172/JCI178692)
Supplement: Supplemental data [file jci-134-178692-s222.pdf]

Supplemental Material

IKZF1 and *UBR4* gene variants drive autoimmunity and TH2 polarization in IgG4-related disease

Qingxiang Liu, Yanyan Zheng, Ines Sturmlechner, Abhinav Jain, Maryam Own, Qiankun Yang, Huimin Zhang, Filippo Pinto e Vairo, Karen Cerosaletti, Jane H. Buckner, Kenneth J. Warrington, Matthew J. Koster, Cornelia M. Weyand, Jörg J. Goronzy

Figure S1

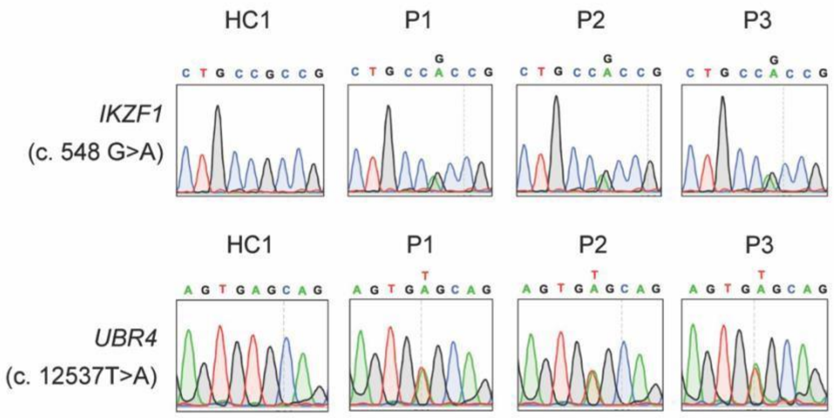

Figure S1. Sanger sequencing validation of *IKZF1* and *UBR4* variants in the patients. Chromatograms from Sanger sequencing from four family members showing the segregation of the G>A variant in *IKZF1* and T>A variant in *UBR4* in the PBMCs of the three affected family members.

Figure S2

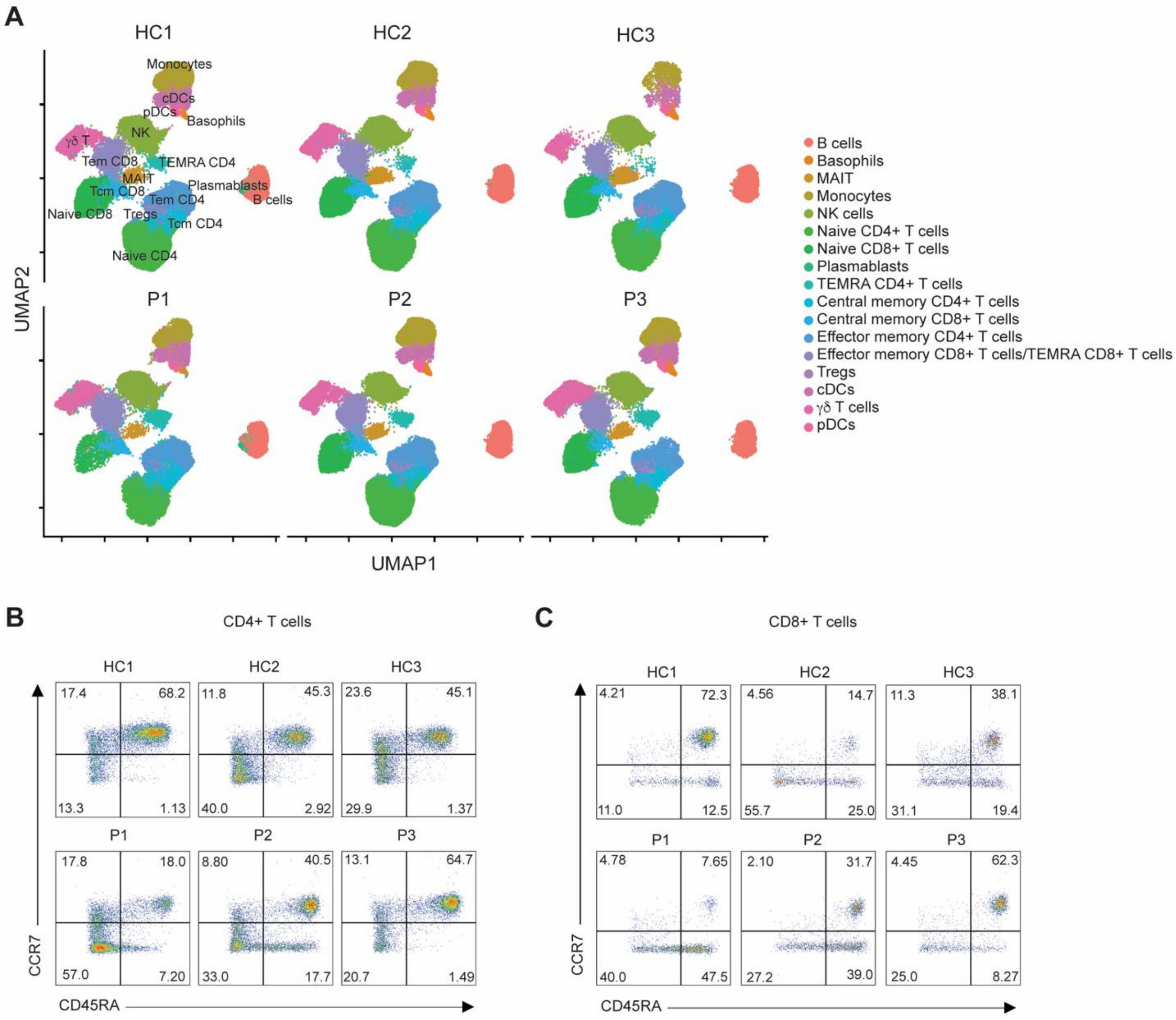

Figure S2. Phenotyping of PBMCs and T cells from patients and healthy controls.

(**A**) UMAP visualization of mass cytometry analysis of individual PBMCs from three healthy controls and three patients.

(**B-C**) Naïve and memory CD4^{+} (**B**) and CD8^{+} (**C**) T cells frequencies determined by CD45RA and CCR7 expression in individual patients and healthy controls. FACS data from HC1 and P1 (Figure 2C) are shown again in the context of data from additional healthy controls and patients.

Figure S3

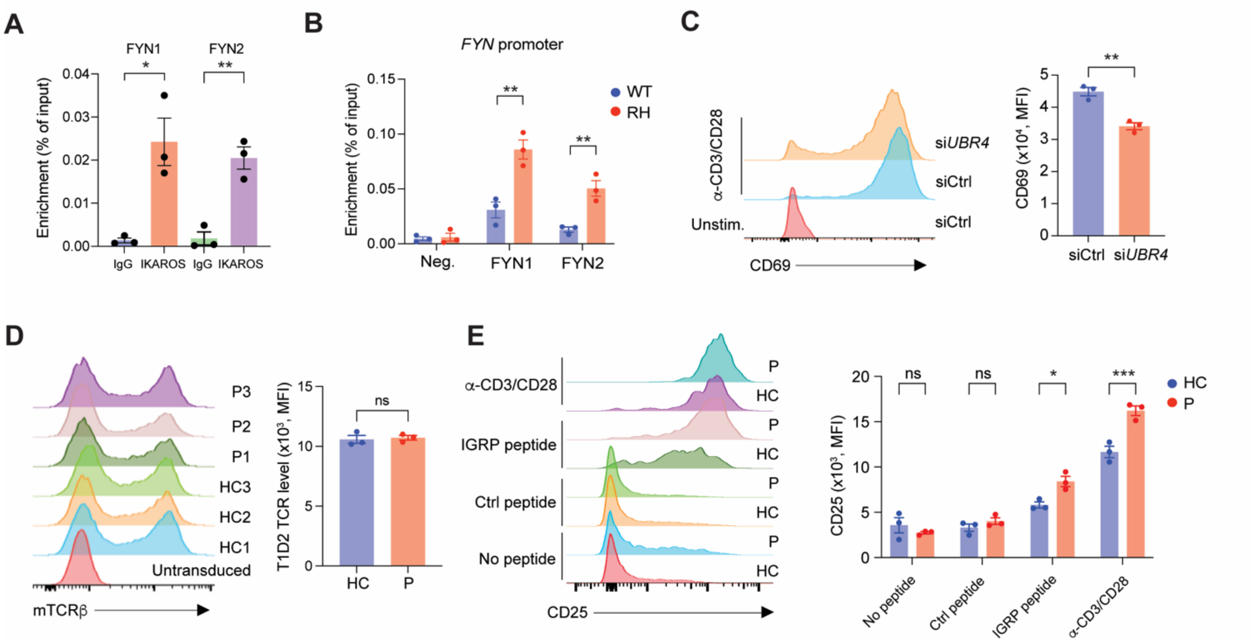

Figure S3. Synergistic roles of IKAROS and UBR4 in causing FYN hyperactivity.

(**A-B**) ChIP-qPCR analysis of IKAROS binding with two *FYN* promoter primer sets. HEK293T cells were transfected with wild type IKAROS (**A**) or with wild type or with R183H *IKFZ1* variant (**B**).

(**C**) CD69 expression measured by flow cytometry on total T cells from healthy individuals transfected with control siRNA or UBR4 siRNA after anti-CD3/CD28 stimulation for 24 hrs.

(**D**) Flow cytometry analysis of the expression of the T1D2 transgenic islet-specific TCR on T cell blasts from patients and healthy controls after lentiviral transduction using anti-mouse TCR β chain antibody.

(**E**) CD69 expression after stimulation with no peptide, control peptide, or IGRP peptide-loaded APC measured by flow cytometry of T1D2 TCR transgenic T cells derived from healthy controls (n=3) and patients (n=3). Anti-CD3/CD28 stimulation served as a positive control.

Data are presented as mean ± s.e.m and were analyzed by two-tailed unpaired Student's t-test (**A-D**) or two-way ANOVA (**E**). *P < 0.05, **P < 0.01, ***P< 0.001. ns, not significant.

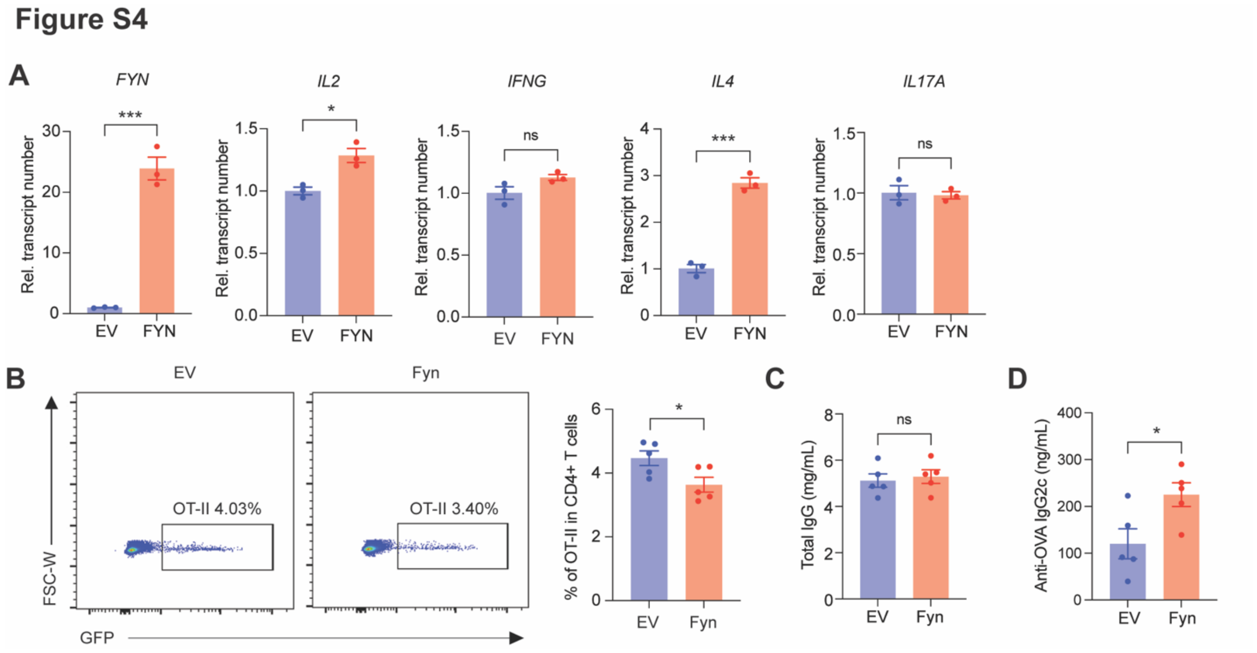

Figure S4. IKAROS-mediated, increased FYN induces TH2 skewing.

(**A**) Naïve CD4 T cells from healthy individuals were lentivirally transduced with empty vector (EV) or vector encoding *FYN*. Cells were cultured on anti-CD3/CD28 coated plates for 2 days under non-polarizing conditions. mRNA expression was analyzed by qRT-PCR.

(**B**) Flow cytometry analysis of splenocytes of the recipient mice. GFP positive population indicates the transduced, transgenic OT-II cells.

(**C**-**D**) Serum total IgG (**C**) and anti-OVA IgG2c (**D**) levels were determined by ELISA.

Data are presented as mean ± s.e.m and were analyzed by two-tailed unpaired Student's t-test. *P < 0.05, ***P< 0.001. ns, not significant.

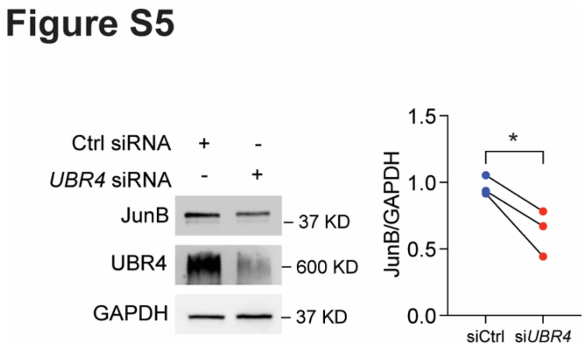

Figure S5. The effect of UBR4 silencing on JunB abundance.

Immunoblot analysis of total T cells from healthy individuals transfected with control siRNA or *UBR4* siRNA after anti-CD3/CD28 stimulation for 24 h.

Data are presented as mean ± s.e.m and were analyzed by two-tailed paired Student's t-test. *P < 0.05.

Figure S6

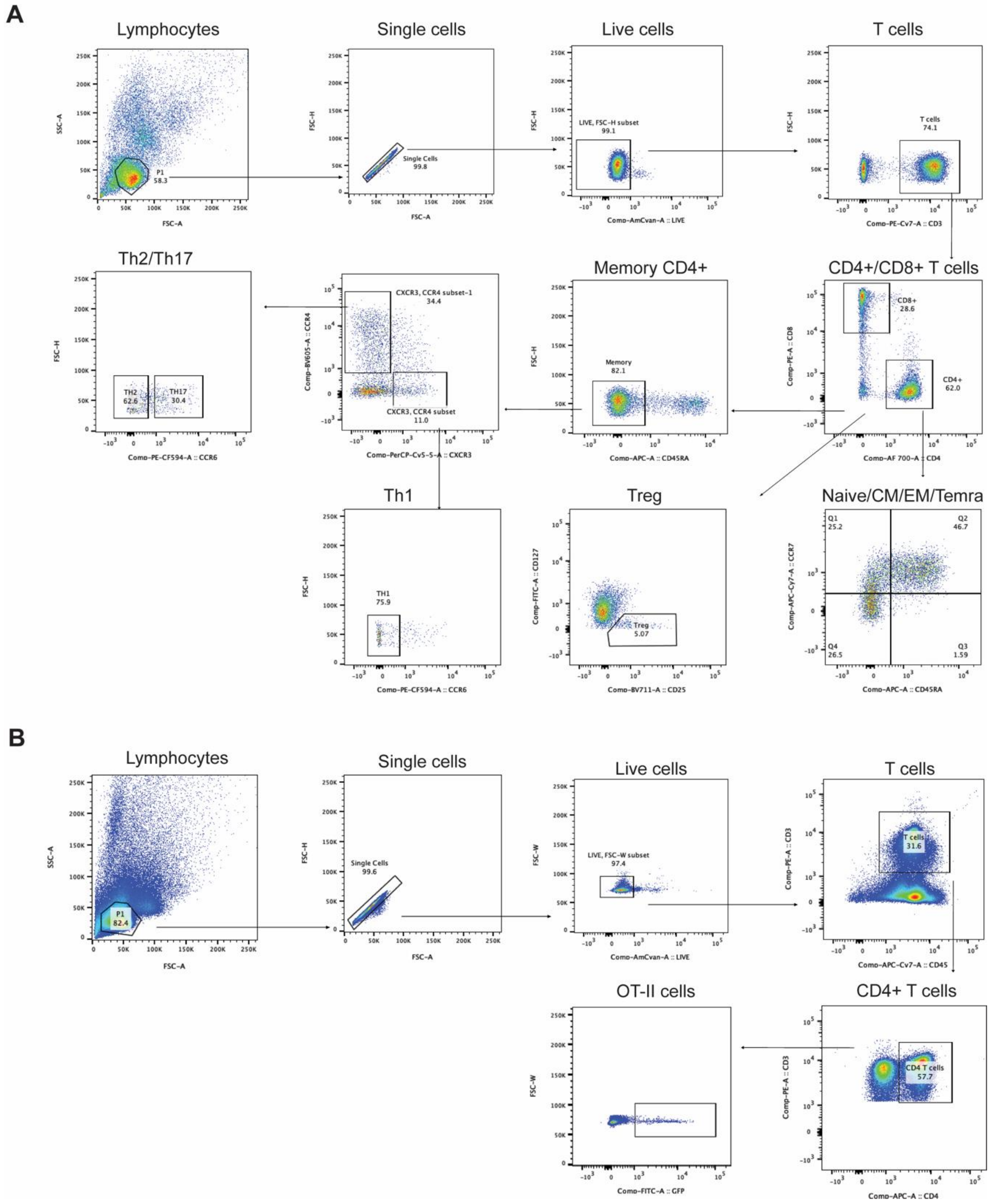

Figure S6. Gating strategies used in this study.

(**A**) Gating strategy for analysis of PBMCs from patients and healthy controls.

(**B**) Gating strategy for analysis of mouse splenocytes.

Table S1. Clinical manifestations of the patients

Patient	Age at first presentation/ evaluation (yrs)	Origin	Autoantibodies	Atopy/ allergy	Lymphadenopathy	Other organ involvement	Treatment
P1 (Father)	12	Russia	Not available	Allergic rhinitis, bronchial wall thickening	Cervical, ~~right~~ axillary, mediastinal, inguinal	Dacryoadenitis orbital masses	Unknown
P2 (Older daughter, index case)	17	Russia	Negative ANA, ANCA, RF	Allergic rhinitis, urticaria, bronchial wall thickening	Neck, chest, abdomen, pelvis, inguinal	Dacryoadenitis, pulmonary ground glass opacities /micronodules	Steroids, Azathioprine, Rituximab
P3 (Sister)	12	Russia	Negative ANA, RF, dsDNA, ENA, ANCA	Allergic rhinitis, urticaria	Cluster of Infra-renal para-aortic lymph nodes with small FDG uptake	None	Never treated

Table S2. Laboratory tests

	P1	P2	P3
**Laboratory test (reference range)**			
IgM mg/dl (45-244)	NA	83	154
IgA mg/dl (52-319)	430	578	559
IgG total mg/dl (767-1590)	1110	2100	991
IgG1 mg/dl (341-894)	NA	1210	NA
IgG2 mg/dl (171-632)	NA	637	NA
IgG3 mg/dl (18-106)	NA	117	NA
IgG4 (<121 mg/dl)	205	486	136
IgE kU/L (<214)	2221	1247	7600
Leukocytes x10^9/L	9.1	8.8	12.4
Neutrophils %	50	78.2	70
Lymphocytes %	35.9	7.8	21.5
Eosinophils %	5.8	4.7	1.7
Hemoglobin g/dl	14.9	12.6	13.7
Platelet x10^9/L	236	352	333
**Red** denotes abnormal laboratory values. **Black** denotes laboratory values within the normal reference range. NA: not assessed.			

Table S3. CyTOF antibody panel

Target	Clone	Metal	Supplier	Catalog #
CD45	HI30	89Y	Fluidigm	S3089003C
CD196/CCR6	G034E3	141Pr	Fluidigm	S3141003C
CD19	HIB19	142Nd	Fluidigm	S3142001C
CD127/IL-7Ra	A019D5	143Nd	Fluidigm	S3143012C
CD38	HIT2	144Nd	Fluidigm	S3144014C
IgD	IA6-2	146Nd	Fluidigm	S3146005C
CD11c	Bu15	147Sm	Fluidigm	S3147008C
CD16	3G8	148Nd	Fluidigm	S3148004C
CD194/CCR4	L291H4	149Sm	Fluidigm	S3149029C
CD123/IL-3R	6H6	151Eu	Fluidigm	S3151001C
TCRgd	11F2	152Sm	Fluidigm	S3152008C
CD185/CXCR5	RF8B2	153Eu	Fluidigm	S3153020C
CD3	UCHT1	154Sm	Fluidigm	S3154003C
CD45RA	HI100	155Gd	Fluidigm	S3155011C
CD27	L128	158Gd	Fluidigm	S3158010C
CD28	CD28.2	160Gd	Fluidigm	S3160003C
CD66b	80H3	162Dy	Fluidigm	S3162023C
CD183/CXCR3	G043H7	163Dy	Fluidigm	S3163004C
CD161	HP-3G10	164Dy	Fluidigm	S3164009C
CD45RO	UCHL1	165Ho	Fluidigm	S3165011C
CD24	ML5	166Er	Fluidigm	S3166007C
CD197/CCR7	G043h7	167Er	Fluidigm	S3167009C
CD8	SK1	168Er	Fluidigm	S3168002C
CD25	2A3	169Tm	Fluidigm	S3169003C
CD20	2H7	171Yb	Fluidigm	S3171012C
HLA-DR	L243	173Yb	Fluidigm	S3173005C
CD4	SK3	174Yb	Fluidigm	S3174004C
CD14	M5E2	175Lu	Fluidigm	S3175015C
CD56/NCAM	NCAM16.2	176Yb	Fluidigm	S3176008C

Table S4. Antibodies for flow cytometry and immunoblotting

Antibody name	Clone	Supplier	Catalog #	Application
Anti-human CD3 PE-Cy7	SK7	BioLegend	300419	Flow cytometry
Anti-human CD4 AF700	SK3	BioLegend	317426	Flow cytometry
Anti-human CD8 PE	SK1	BioLegend	980902	Flow cytometry
Anti-human CCR7 AF750	G043H7	BioLegend	353245	Flow cytometry
Anti-human CD127 FITC	A019D5	BioLegend	351311	Flow cytometry
Anti-human CD25 BV711	BC96	BioLegend	302635	Flow cytometry
Anti-human CXCR3 PerCP-Cy5.5	G025H7	BioLegend	353714	Flow cytometry
Anti-human CXCR5 BV421	J252D4	BioLegend	356919	Flow cytometry
Anti-human CCR4 BV605	L291H4	BioLegend	359417	Flow cytometry
Anti-human CCR6 PE-CF594	G034E3	BioLegend	353429	Flow cytometry
Anti-human CD45 APC	HI130	BioLegend	304011	Flow cytometry
Anti-human CD45RA APC	HI100	BioLegend	304112	Flow cytometry
Anti-phospho-ERK AF488	20A	BD	612592	Flow cytometry
Anti-human CD69 APC	FN50	BioLegend	310910	Flow cytometry
Anti-human CD25 BV711	BC96	BioLegend	302635	Flow cytometry
Anti-phospho-SYK Y525/526 AF647	C87C1	Cell Signaling	12081	Flow cytometry
Anti-human IL-4 PE	8D4-8	Invitrogen	12-7049-41	Flow cytometry
Anti-mouse IL-4 BV711	11B11	BioLegend	504133	Flow cytometry
Anti-mouse IFNγ PE-Cy7	XMG1.2	BD	557649	Flow cytometry
Anti-mouse TCR β chain PerCP-Cy5.5	H57-597	BioLegend	109227	Flow cytometry
Anti-CD45	D9M8I	Cell Signaling	13917	Immunoblot
Anti-Phospho-LCK Y394	755103	R&D Systems	MAB7500	Immunoblot
Anti-Phosph-LCK Y505	Polyclonal	Cell Signaling	2751	Immunoblot
Anti-LCK	Polyclonal	Cell Signaling	2752	Immunoblot
Anti-CSK	C74C1	Cell Signaling	4980	Immunoblot
Anti-Phospho-ZAP70 Y319	65E4	Cell Signaling	2717	Immunoblot
Anti-ZAP70	99F2	Cell Signaling	2705	Immunoblot
Anti-NUR77	EPR23982-85	Abcam	ab283264	Immunoblot
Anti-LYN	C13F9	Cell Signaling	2796	Immunoblot
Anti-FYN	Polyclonal	Cell Signaling	4023	Immunoblot
Anti-FYN	1S	Invitrogen	MA5-13134	Co-IP
Anti-Phospho-SRC Y527	Polyclonal	Cell Signaling	2105	Immunoblot
Anti-IKAROS	D10E5	Cell Signaling	9034	Immunoblot, ChIP
Anti-UBR4	Polyclonal	Abcam	Ab86738	Immunoblot
Anti-Phospho-ERK	D13.14.4E	Cell Signaling	4370	Immunoblot
Anti-ERK	137F5	Cell Signaling	4695	Immunoblot
Anti-His tag	Polyclonal	Cell Signaling	2365	Immunoblot
Anti-JunB	C37F9	Cell Signaling	3753	Immunoblot, co-IP
Anti-Ubiquitin HRP	P4D1	Cell Signaling	14049	Immunoblot
Anti-GAPDH	D16H11	Cell Signaling	8884	Immunoblot
Anti-β-actin HRP	8H10D10	Cell Signaling	12262	Immunoblot
Anti-Rabbit IgG HRP	Polyclonal	Cell Signaling	7074	Immunoblot
Anti-Rabbit IgG (Confirmation specific) HRP	L27A9	Cell Signaling	5127	Immunoblot

Table S5. Primers for qRT-PCR

Name	Sequence (5'-3')
PTPRC-F	CTTCAGTGGTCCCATTGTGGTG
PTPRC-R	CCACTTTGTTCTCGGCTTCCAG
FYN-F	ATGGGCTGTGTGCAATGTAAG
FYN-R	GAAGCTGGGGTAGTGCTGAG
TBX21-F	CGGATGTTCCCATTCCTGTC
TBX21-R	TTCCACACTGCACCCACTTG
GATA3-F	GCCCCTCATTAAGCCCAAG
GATA3-R	TTGTGGTGGTCTGACAGTTCG
RORC-F	TGCCAGAATGACCAGATTGTG
RORC-R	ATGCCACCGTATTTGCCTTC
FOXP3-F	CAGCCATGATCAGCCTCACA
FOXP3-R	CACTGGGATTTGGGAAGGTG
IFNG-F	ACTAGGCAGCCAACCTAAGCAAGA
IFNG-R	CATCAGGGTCACCTGACACATTCA
IL4-F	CCAACTGCTTCCCCCTCTG
IL4-R	TCTGTTACGGTCAACTCGGTG
IL2-F	AGAACTCAAACCTCTGGAGGAAG
IL2-R	GCTGTCTCAGCATATTCACAC
IL5-F	TGGAGCTGCCTACGTGTATG
IL5-R	TTCGATGAGTAGAAAGCAGTGC
IL13-F	CCTCATGGCGCTTTTGTTGAC
IL13-R	TCTGGTTCTGGGTGATGTTGA
IL17A-F	TCCCACGAAATCCAGGATGC
IL17A-R	GGATGTTCAGGTTGACCATCAC
RPL13A-F	GCCATCGTGGCTAAACAGGTA
RPL13A-R	GTTGGTGTTCATCCGCTTGC
Il4-F	GGTCTCAACCCCCAGCTAGT
Il4-R	GCCGATGATCTCTCTCAAGTGAT
Il13-F	CCTGGCTCTTGCTTGCCTT
Il13-R	GGTCTTGTGTGATGTTGCTCA
Gata3-F	CTCGGCCATTCGTACATGGAA
Gata3-R	GGATACCTCTGCACCGTAGC
Gapdh-F	AGGTCGGTGTGAACGGATTTG
Gapdh-R	TGTAGACCATGTAGTTGAGGTCA
